# dbGaPCheckup: pre-submission checks of dbGaP-formatted subject phenotype files

**DOI:** 10.1186/s12859-023-05200-8

**Published:** 2023-03-03

**Authors:** Lacey W. Heinsberg, Daniel E. Weeks

**Affiliations:** 1 Department of Human Genetics, School of Public Health, University of Pittsburgh, Public Health 3119, 130 DeSoto Street, Pittsburgh, PA 15261 USA; 2 Department of Biostatistics, School of Public Health, University of Pittsburgh, Public Health 3119, 130 DeSoto Street, Pittsburgh, PA 15261 USA

**Keywords:** Database architecture, Data quality control, Data sharing, Data distribution, Repository, Database of genotypes and phenotypes

## Abstract

**Background:**

Data archiving and distribution are essential to scientific rigor and reproducibility of research. The National Center for Biotechnology Information’s Database of Genotypes and Phenotypes (dbGaP) is a public repository for scientific data sharing. To support curation of thousands of complex data sets, dbGaP has detailed submission instructions that investigators must follow when archiving their data.

**Results:**

We developed dbGaPCheckup, an R package which implements a series of check, awareness, reporting, and utility functions to support data integrity and proper formatting of the subject phenotype data set and data dictionary prior to dbGaP submission. For example, as a tool, dbGaPCheckup ensures that the data dictionary contains all fields required by dbGaP, and additional fields required by dbGaPCheckup; the number and names of variables match between the data set and data dictionary; there are no duplicated variable names or descriptions; observed data values are not more extreme than the logical minimum and maximum values stated in the data dictionary; and more. The package also includes functions that implement a series of minor/scalable fixes when errors are detected (e.g., a function to reorder the variables in the data dictionary to match the order listed in the data set). Finally, we also include reporting functions that produce graphical and textual descriptives of the data to further reduce the likelihood of data integrity issues. The dbGaPCheckup R package is available on CRAN (https://CRAN.R-project.org/package=dbGaPCheckup) and developed on GitHub (https://github.com/lwheinsberg/dbGaPCheckup).

**Conclusion:**

dbGaPCheckup is an innovative assistive and timesaving tool that fills an important gap for researchers by making dbGaP submission of large and complex data sets less error prone.

**Supplementary Information:**

The online version contains supplementary material available at 10.1186/s12859-023-05200-8.

## Background

Making research data publicly available is important to both promote scientific rigor/reproducibility and preserve data beyond the life of the study through which it was originally generated. In recent years, many major funding agencies, including the National Institutes of Health (NIH), have moved toward requiring data sharing across scientific disciplines. A variety of publicly available data repositories exist to facilitate this, including the Database of Genotypes and Phenotypes (dbGaP) [1, 2] which is dedicated to preserving and sharing data from research studies that have collected genetic/genomic data. For example, whole genome sequencing data and rich phenotype data collected through the Jackson Heart Study—a large, community-based, observational study of cardiovascular disease—are stored in dbGaP under the accession number phs000286.v6.p2. To support curation of thousands of complex data sets, dbGaP has very detailed submission instructions [3]. To help researchers meet these multifaceted formatting requirements, and to further support data integrity, we developed dbGaPCheckup, an R package which implements a series of check, reporting, awareness, and utility functions. The package is publicly available at https://CRAN.R-project.org/package=dbGaPCheckup and https://github.com/lwheinsberg/dbGaPCheckup.

## Implementation

This package focuses on two required dbGaP subject phenotype files: (1) the data set, which contains the study data for participants, with each row representing a participant, and each column representing a phenotype variable; and (2) the corresponding data dictionary, which contains descriptions of the variables in the data set with each row representing a unique variable (corresponding to the columns in the data set) and each column representing information about that variable (e.g., variable description, type, minimum/maximum, etc.). For the data dictionary, specifically, dbGaP submission requirements state that, at a minimum, the variable name (VARNAME), description (VARDESC), units (UNITS), and value = meaning (VALUES) columns (e.g., 0 = no, 1 = yes) must be present to facilitate public use of a data set. The dbGaPCheckup package expands on this by requiring additional fields of variable type (TYPE), logical minimum value (MIN), and logical maximum values (MAX) be present to facilitate a series of additional checks.

**Fig. 1:**
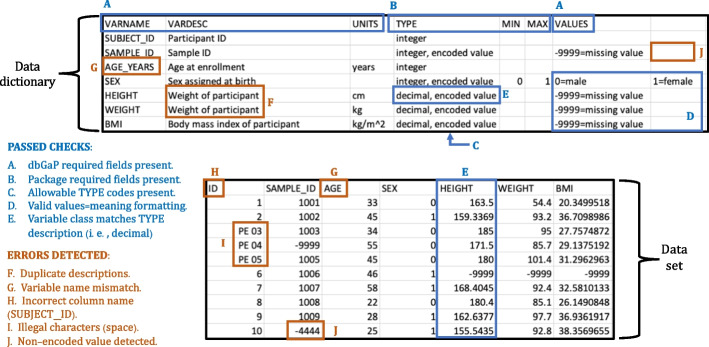
Exemplar dbGaPCheckup pre-submission checks

The functions in our package cover a range of formatting and data integrity checks, many of which are illustrated in Fig. 1 and further detailed in Additional file 1. For example, our checks ensure that the dbGaP-required and package-required data dictionary fields (described above) are present; the number and names of variables match between the data set and data dictionary; observed data values are not more extreme than the logical minimum and maximum values stated in the data dictionary; and more. Complete descriptions (Additional file 1, Section 3) and examples (Additional file 1, Section 6) of the package functions are available in the supplementary information and in our online package documentation at https://lwheinsberg.github.io/dbGaPCheckup/index.html. To initiate the package, users simply load the data set and data dictionary and call the check_report() function (Additional file 1, Section 6.1.1) which implements a panel of dbGaPCheckup checks.

Beyond the formatting and data checks described above, dbGaPCheckup includes several helper functions that implement minor/scalable fixes. For example, if full application of all available checks is limited because the input data dictionary is missing the dbGaP-optional but package-required data dictionary fields of TYPE, MIN, and MAX, they can easily be added using the add_missing_fields() function (Additional file 1, Section 6.1.1) which infers TYPE from the data and simply adds MIN/MAX as empty columns (as the “logical” minimum and maximum values for a given study can only truly be known by the study investigators themselves). Similarly, the reorder_dictionary() function (Additional file 1, Section 6.1.5) reorders the variables in the data dictionary to match the order listed in the data set. Most problems the package identifies, however, will need to be manually corrected by the user as issues are most often study-specific (e.g., coding mismatches; typographical errors; etc.).

Our package also includes reporting functions (i.e., create_report() and create_awareness_report(); Additional file 1, Sections 6.2 and 7) that generate graphical and textual descriptives of the data to support more detailed and subjective interrogation. The creation of these report functions was motivated by challenges of (1) checking for consistent use of user-defined missing value codes (e.g., − 4444, − 9999) and (2) attempting to check for missing value = meaning map elements in dbGaP’s unique format (e.g., − 9999 = missing value; 0 = no).

Finally, we have created the label_data() function (Additional file 1, Section 6.3) which can be utilized to add information from the data dictionary as attributes to the data set stored as a unified R data frame for ease of future use [4, 5]. This function even enables SPSS-style encoding/handling of user-defined missing value codes [4, 5]. Once data are labelled, variable names and codes are automatically displayed within certain views of the data, leading to more human-interpretable and less error-prone workflows and analyses.

## Discussion and conclusions

Our R package, dbGaPCheckup, is an innovative assistive and timesaving tool that fills an important gap for NIH researchers as it will make dbGaP submission of large and complex data sets less error prone. In fact, through both the objective checks, as well as the more subjective awareness reports, we have identified pre-submission errors in our own data sets that we likely would not have been aware of otherwise. Not only is our package simple-to-use but it also eases the burden of complying with the many dbGaP formatting requirements. Further, in our own prior workflows and collaborations, we have found that having the data set and data dictionary as separate files adds a modicum of difficulty in looking up information in the data dictionary; unfortunately, even small hurdles such as this is sometimes deterring in looking up required information. The label_data() function, in particular, brings huge advantages in addressing this issue by merging the data dictionary and data set so that the data dictionary information moves with the data and is readily and easily available.

To our knowledge, the only other available dbGaP data integrity software is the National Center for Biotechnology Information’s GaPTools [6]. Compared to dbGaPCheckup’s more targeted focus on the phenotype data set and data dictionary, GaPTools implements checks for a broader variety of required dbGaP files such as those housing data for the sample attributes, subject sample mapping, pedigrees/genotypes (if relevant), and consents, making it an important tool in the data submission process. For the phenotype data files, specifically, however, the current release of GaPTools implements only a subset of dbGaPCheckup checks. For example, while GaPTools does flag issues such as missing required data dictionary fields, variables that are listed in one subject phenotype data file but not the other, or variables with missing data dictionary descriptions, it does not currently flag duplicated variable descriptions, values that fall outside of the listed logical minimum/maximum value ranges, inaccurate variable types, or missing value = meaning codes. Further, GaPTools requires Docker installation/knowledge of command line workflows and is currently not optimized for fast iterative and interactive use, so therefore does not host any helper utilities (e.g., reorder_dictionary()). Finally, GaPTools does not produce reports like those provided by dbGaPCheckup which enable one to better understand and visualize study data. Given the streamlined and easy-to-use format of dbGaPCheckup, and its more comprehensive set of checks for the phenotype data set and data dictionary, specifically, we recommend that it be used during the iterative phenotype data curation phase and, once the broader final suite of files has been set up, complemented by the GaPTools pre-validation software just prior to dbGaP submission.

Beyond its original intended purpose, dbGaPCheckup provides important extrinsic merit. Given the latest data sharing policy from the NIH, which require all researchers (and not just those generating large scale genomic data) to make their data publicly available as of January 2023 [8], we believe universal adoption of dbGaP formatting and database management styles should be considered by all academic institutions and investigators for database harmonization. A standardized database format of this nature would not only make submissions to data repositories simpler and faster, but would also make internal data management, curation, merging, and sharing across research groups easier. If such an approach is taken, dbGaPCheckup will have important utility across the database architecture phases preceding data submission, further supporting NIH researchers during this important transition into an era of more rigorous and reproducible science.

## Supplementary Information

**Additional file 1**. is a PDF file adapted from the dbGaPCheckup vignette. It contains additional information about dbGaP subject phenotype files and submission instructions, how dbGaPCheckup can be used as a curation tool during the pre-submission phase, and detailed applied examples of dbGaPCheckup including discussion of function input, output, and interpretation.

## Data Availability

Software name: dbGaPCheckup. Software home page: https://lwheinsberg.github.io/dbGaPCheckup/index.html; https://CRAN.R-project.org/package=dbGaPCheckup; https://github.com/lwheinsberg/dbGaPCheckup. Other relevant links: An academic archive of the dbGaPCheckup package code from February 14, 2023 (date of manuscript revision submission) is available in Zenodo under https://doi.org/10.5281/zenodo.7640426. Operating system(s): Linux, Mac, Windows. Programming language: R. License: GPL-2.

## Abbreviations

NIH: National Institutes of Health
dbGaP: Database of Genotypes and Phenotypes
