# Supplementary material for: dbGaPCheckup: pre-submission checks of dbGaP-formatted subject phenotype files
Source: BMC Bioinformatics. 2023 Mar 3;24:77. doi: 10.1186/s12859-023-05200-8 (PMC9985192; doi:10.1186/s12859-023-05200-8)
Supplement: Supplementary file 1 — Additional file 1. is a PDF file adapted from the dbGaPCheckup vignette. It contains additional information about dbGaP subject phenotype files and submission instructions, how dbGaPCheckup can be used as a curation tool during the pre-submission phase, and detailed applied examples of dbGaPCheckup including discussion of function input, output, and interpretation. [file 12859_2023_5200_MOESM1_ESM.pdf]

Supplementary Information
for
dbGaPCheckup: pre-submission checks of dbGaP-formatted subject phenotype files

Lacey W. Heinsberg and Daniel E. Weeks
University of Pittsburgh School of Public Health

Contents

1 Introduction **2**

2 Software copyright information **2**

3 Overview **2**

4 Installation **4**

5 Data format, file types, and file names **4**

5.1 Files . . . 4
5.2 Subject Phenotype Data Set (DS) . . . 5
5.3 Subject Phenotype Data Dictionary (DD) . . . 5

6 Execution with example runs and interpretation **6**

6.1 Check, utility, and awareness functions . . . 6
6.1.1 Example 1 . . . 6
6.1.2 Example 2 . . . 9
6.1.3 Example 3 . . . 14
6.1.4 Example 4 . . . 18
6.1.5 Example 5 . . . 19
6.1.6 Example 6 . . . 23
6.2 Reporting functions . . . 24
6.3 Label data function . . . 25

7 Appendix: Reporting functions **25**

7.1 Appendix A: Awareness Report 25

7.1.1 Missingness Summary 26

7.1.2 Values Missing Tables 27

7.1.2.1 Check A: If the user defines a missing value code that is not present in the data (In Set M and Not in Set D). 28

7.1.2.2 Check B: If a VALUES entry defines an encoded code value, but that value is not present in the data (In Set V and Not in Set D). 28

7.1.2.3 Check C: If the user defines a missing value code that is not defined in a VALUES entry (In Set M and Not in Set V). 30

7.1.2.4 Check D: If a user-defined missing value code is present in the data for a given variable, but that variable does not have a corresponding VALUES entry (M in Set D and Not in Set V). 30

7.1.2.5 Check E: If a VALUES entry is NOT defined as a missing value code AND is NOT identified in the data. ((Set V values that are NOT in Set M) that are NOT in Set D). 31

7.2 Appendix B: Data Report 31

7.2.1 Summary and plots 31

8 Contact information **48**

9 Acknowledgments **49**

1 Introduction

This document describes our R package, `dbGaPCheckup`, and was adapted from the February 2023 `dbGaPCheckup` vignette for use as Supplementary Information to our manuscript entitled dbGaPCheckup: pre-submission checks of dbGaP-formatted subject phenotype files. Online versions of our package documentation can be found at: https://github.com/lwheinsberg/dbGaPCheckup and https://lwheinsberg.github.io/dbGaPCheckup/. An archive of the dbGaPCheckup package at the date of final manuscript submission can be found at Zenodo under doi `10.5281/zenodo.7640426`.

2 Software copyright information

Copyright 2022, University of Pittsburgh. All Rights Reserved. License: GPL-2

3 Overview

`dbGaPCheckup` implements a series of check, awareness, utility, and reporting functions to help you ensure your scientific data set meets formatting requirements for submission to the National Center for Biotechnology Information's database of Genotypes and Phenotypes (dbGaP). This document was designed to provide a broad overview of the utility of this R package. A complete table of functions and descriptions is shown below.

Table 1: List of function names and types.

Function_Name	Function_Type	Function_Description
field_check	check	Checks for dbGaP required fields: variable name (VARNAME), variable description (VARDESC), units (UNITS), and variable value and meaning (VALUES).
pkg_field_check	check	Checks for package-level required fields: variable type (TYPE), minimum value (MIN), and maximum value (MAX).
dimension_check	check	Checks that the number of variables match between the data set and data dictionary.
name_check	check	Checks that variable names match between the data set and data dictionary.
id_check	check	Checks that the first column of the data set is the primary ID for each participant labeled as SUBJECT_ID, that values contain no illegal characters or padded zeros, and that each participant has an ID.
row_check	check	Checks for empty or duplicate rows in the data set and data dictionary.
NA_check	check	Checks for NA values in the data set and, if NA values are present, also checks for an encoded NA value=meaning description.
type_check	check	If a TYPE field exists, this function checks for any TYPE entries that aren't allowable per dbGaP instructions.
values_check	check	Checks for potential errors in the VALUES columns by ensuring (1) required format of ‘VALUE=MEANING‘ (e.g., 0=No or 1=Yes); (2) no leading/trailing spaces near the equals sign (e.g., 0=No vs. 0 = No); (3) all variables of TYPE encoded have VALUES entries; and (4) all variables with VALUES entries are listed as TYPE encoded.
integer_check	check	Checks for variables that appear to be incorrectly listed as TYPE integer.
decimal_check	check	Checks for variables that appear to be incorrectly listed as TYPE decimal.
misc_format_check	check	Checks miscellaneous dbGaP formatting requirements to ensure (1) no empty variable names; (2) no duplicate variable names; (3) variable names do not contain "dbgap"; (4) there are no duplicate column names in the dictionary; and (5) column names falling after ‘VALUES‘ column are unnamed.
description_check	check	Checks for unique and non-missing descriptions (VARDESC) for every variable in the data dictionary.
minmax_check	check	Checks for variables that have values exceeding the listed MIN or MAX.
missing_value_check	check	Checks for variables that have non-encoded missing value codes.
complete_check	bulk check	Runs the entire workflow (field_check, pkg_field_check, dimension_check, name_check, id_check, row_check, NA_check, type_check, values_check, integer_check, decimal_check, misc_format_check, description_check, minmax_check, and missing_value_check).
add_missing_fields	utility	Adds additional fields required by this package including variable type ('TYPE'), minimum value ('MIN'), and maximum value ('MAX').
name_correct	utility	Updates the data set so variable names match those listed in the data dictionary.
reorder_dictionary	utility	Reorders the data dictionary to match the data set.
reorder_data	utility	Reorders the data set to match the data dictionary.
id_first_data	utility	Reorders the data set so that SUBJECT_ID comes first.
id_first_dict	utility	Reorders the data dictionary so that SUBJECT_ID comes first.
label_data	utility, awareness	Adds non-missing information from the data dictionary as attributes to the data.
value_meaning_table	utility, awareness	Generates a value-meaning table by parsing the VALUES fields.
missingness_summary	awareness	Summarizes the amount of missingness in the data set.
value_missing_table	awareness	Checks for consistent usage of encoded values and missing value codes between the data dictionary and the data set.
dictionary_search	awareness	Facilitates searches of the data dictionary.
check_report	bulk check, reporting	Generates a user-readable report of the checks run by the complete_check function.
create_report	reporting, awareness	Generates a textual and graphical report of the selected variables in HTML format.
create_awareness_report	reporting, awareness	Generates an awareness report, calling missingness_summary and value_missing_table functions.

4 Installation

The package is written in R language.

To install from CRAN, proceed as follows:

```
install.packages("dbGaPCheckup")
```

To install the development version from GitHub, proceed as follows:

1. Install and load the `devtools` package by issuing these commands:

```
install.packages("devtools")
library(devtools)
```

2. Install and load the `dbGaPCheckup` by issuing these commands:

```
install_github("lwheinsberg/dbGaPCheckup/pkg")
```

If you wish to have this vignette installed and accessible within your R help pages, use this command instead:

```
install_github("lwheinsberg/dbGaPCheckup/pkg", build_opts = c("--no-resave-data", "--no-manual"),
build_vignettes = TRUE)
```

After the `dbGaPCheckup` package has been installed, you can view load the package using this command:

```
library(dbGaPCheckup)
```

and view this vignette using:

```
browseVignettes("dbGaPCheckup")
```

5 Data format, file types, and file names

dbGaP has a host of formatting requirements for data set submission.

This package focuses on two required files: the Subject Phenotype data set (DS) and the corresponding Subject Phenotype data dictionary (DD). Brief instructions on setting up the files have been included below.

5.1 Files

Checks that are NOT currently embedded into this package that we want to draw special attention to include:

(1) You may ONLY submit tab-delimited .txt and .xlsx files.
–> Tab-delimited txt files are preferable for the data set.
–> Excel (.xlsx) format is preferable for the data dictionary.

(2) File names should NOT contain special characters, spaces, hyphens, brackets, periods, or forward (/) or backward slashes ().
–> For example, 'data.set.txt', 'data-set.txt', 'data set.txt' are all illegal names, but 'data_set.txt' would be OK.

(3) Excel files are only allowed to have one sheet (i.e., no multiple tabs/sheets are allowed).

5.2 Subject Phenotype Data Set (DS)

In brief, the Subject Phenotype data set consists of the study data for participants. In the data set, each row represents a participant, and each column represents a study phenotype variable. The first column in the data set needs to be labeled `SUBJECT_ID` and contains the unique participant identifier as an integer or string value. Integers should not have zero padding or spaces. Specifically, only the following characters can be included in the ID: English letters, Arabic numerals, period (.), hyphen (-), underscore (_), at symbol (@), and the pound sign (#). Columns falling after `SUBJECT_ID` will be unique to a given study, but include participant factors such as age, sex, etc. Formatting for an example data set is shown below.

Table 2: First six rows and columns of an example dbGaP data set.

SUBJECT_ID	SAMPLE_ID	AGE	SEX	PREGNANT	HEIGHT
1	1001	33	0	-4444	163.5
2	1002	45	1	0	159.3
3	1003	34	0	-4444	185
4	-9999	55	0	-4444	171.5
5	1005	45	0	-4444	180
6	1006	46	1	0	-9999

Other example data sets provided by dbGaP can be found at the NCBI submission guide. See "Example of a Subject Phenotypes DS File" and "6a_SampleAttributes_DS.txt".

5.3 Subject Phenotype Data Dictionary (DD)

In the Subject Phenotype data dictionary, each row represents a unique variable (that corresponds to columns in the data set), and each column represents information about that variable (see example below). For more detailed data dictionary formatting instructions, visit the NCBI submission guide and see heading "APPENDIX for Data Dictionary (DD) File Descriptions and Specifications", which includes a table of required and suggested column headers and descriptions, as well as an example file called "6b_SampleAttributes_DD.xlsx".

Table 3: First five rows and nine columns of an example dbGaP data dictionary. (continued below)

VARNAME	VARDESC	TYPE	UNITS
SUBJECT_ID	Participant ID	integer	NA
SAMPLE_ID	Sample ID	integer, encoded value	NA
AGE	Age at enrollment	integer	years
SEX	Sex assigned at birth	integer, encoded value	NA
PREGNANT	Pregnancy status at enrollment	integer, encoded value	NA

MIN	MAX	VALUES	...18	...19
NA	NA	NA	NA	NA
NA	NA	-9999=missing value	NA	NA
NA	NA	NA	NA	NA
0	1	0=male	1=female	NA
0	1	0=no	1=yes	-9999=missing value

Two special data dictionary formatting notes:

(1) The final columns of the data dictionary list all unique values/meanings of all encoded values, one value per cell, of which will vary based on the number of VALUE codes for a specific variable. For example, if your data set contains a variable called `SEX` in which 0 indicates female and 1 indicates male, these columns are designed to communicate `value=meaning` (e.g., 0=female). The `VALUES` header must be the last column header and should appear ONLY in the column above the FIRST encoded value that is listed. The remaining value column header cells should be left blank. (Note that when we read in our example data set with blank column names after `VALUES`, R automatically fills in the column names with the column number (e.g., `...18`, `...19`, etc.). This is acceptable for the package level checks, but not allowable for the files that are submitted to dbGaP.)

(2) This package requires several fields beyond those required by the dbGaP formatting requirements to support our additional data integrity checks. Specifically, dbGaP requires only that the data dictionary contains the following fields: variable name (`VARNAME`); variable description (`VARDESC`); units (`UNITS`); and variable value and meaning (`VALUE`). Because this package was designed to perform both dbGaP formatting requirement checks, as well as a series of awareness checks to help you ensure data accuracy, this package also requires that the data dictionary contains the following additional fields: logical minimum (`MIN`) and logical maximum (`MAX`) values (allowed to be left blank, but column headers are required) and the data type (e.g., integer, decimal, encoded value, string; `TYPE`) fields. If your data dictionary does not include these additional fields already, you can simply use the `add_missing_fields` function to auto fill them (see below).

6 Execution with example runs and interpretation

6.1 Check, utility, and awareness functions

Note that all "check" functions included in our package return an invisible tibble that contains (1) Time (Time stamp); (2) Name (Name of the function); (3) Status (Passed/Failed); (4) Message (A copy of the message the function printed out); and (5) Information (More detailed information about the potential errors identified). This was designed to streamline the complete workflow approach and to return a succinct report back to you via `check_report` (see below). Note that there are some dependencies between checks (e.g., name_check `values_check` is dependent upon `field_check`), so there are pre-checks embedded within many checks.

6.1.1 Example 1

```
data(ExampleD)
```

We recommend starting with the `check_report` function, which includes 15 embedded checks. Note that for all functions, you need to first specify the name of the data dictionary, followed by the name of the data set.

```
e1_report <- check_report(DD.dict.D, DS.data.D, non.NA.missing.codes=c(-4444, -9999))
#> # A tibble: 15 x 3
#>    Function            Status        Message
#>    <chr>               <chr>         <chr>
#>  1 field_check         Passed        Passed: required fields VARNAME, VARDESC, ~
#>  2 pkg_field_check     Failed        ERROR: not all package-level required fiel~
#>  3 dimension_check     Passed        Passed: the variable count matches between~
```

```
#>  4 name_check          Passed        Passed: the variable names match between t~
#>  5 id_check            Passed        Passed: All ID variable checks passed.
#>  6 row_check           Passed        Passed: no blank or duplicate rows detecte~
#>  7 NA_check            Not attempted ERROR: Required pre-check pkg_field_check ~
#>  8 type_check          Failed        ERROR: TYPE column not found. Consider usi~
#>  9 values_check        Not attempted ERROR: Required pre-check type_check faile~
#> 10 integer_check       Not attempted ERROR: Required pre-check pkg_field_check ~
#> 11 decimal_check       Not attempted ERROR: Required pre-check pkg_field_check ~
#> 12 misc_format_check   Passed        Passed: no check-specific formatting issue~
#> 13 description_check   Failed        ERROR: missing and duplicate descriptions ~
#> 14 minmax_check        Not attempted ERROR: Required pre-check pkg_field_check ~
#> 15 missing_value_check Not attempted ERROR: Required pre-check pkg_field_check ~
#> --------------------
#> pkg_field_check: Failed
#> ERROR: not all package-level required fields are present in the data dictionary.
↪  Consider using the add_missing_fields function to auto fill these fields.
#> $pkg_field_check.Info
#>  TYPE   MIN   MAX
#> FALSE FALSE FALSE
#>
#> --------------------
#> type_check: Failed
#> ERROR: TYPE column not found. Consider using the add_missing_fields function to
↪  autofill TYPE.
#> $type_check.Info
#> [1] "ERROR: TYPE column not found."
#>
#> --------------------
#> description_check: Failed
#> ERROR: missing and duplicate descriptions found in data dictionary.
#> $description_check.Info
#> # A tibble: 4 x 2
#>   VARNAME  VARDESC
#>   <chr>    <chr>
#> 1 PREGNANT <NA>
#> 2 REACT    <NA>
#> 3 HEIGHT   Height of participant
#> 4 WEIGHT   Height of participant
#>
#> --------------------
```

In this check, we see that several checks passed (e.g., `field_check`), some failed (e.g., `type_check`), and some could not be attempted because a pre-check in the function failed (e.g., `missing_value_check`).

The `check_report` output can be examined to better understand the issues at hand. For example, let's examine the `pkg_field_check` results more closely. You can call more detailed information for each check using the following commands:

```
e1_report$Message[2]
#> [1] "ERROR: not all package-level required fields are present in the data dictionary.
↪  Consider using the add_missing_fields function to auto fill these fields."
e1_report$Information$pkg_field_check.Info
#>  TYPE   MIN   MAX
#> FALSE FALSE FALSE
```

Here, we see that the `TYPE`, `MIN`, and `MAX` columns required for the complete workflow approach in this package are missing. But never fear - we can simply use the `add_missing_fields` function to add these in!

```
DD.dict_updated <- add_missing_fields(DD.dict.D, DS.data.D)
#> $Message
#> [1] "CORRECTED ERROR: not all package-level required fields were present in the data
↪  dictionary. The missing fields have now been added! TYPE was inferred from the data,
↪  and MIN/MAX have been added as empty fields."
#>
#> $Missing
#> [1] "TYPE" "MIN"  "MAX"
```

Now that our error has been corrected, let's return to `check_report` to further investigate. Don't forget to call in the updated version of the data dictionary here!

```
# Note! Don't forget to call in the updated version of the data dictionary here!
e1_report.v2 <- check_report(DD.dict_updated, DS.data.D,
          non.NA.missing.codes=c(-4444, -9999))
#> # A tibble: 15 x 3
#>    Function            Status Message
#>    <chr>               <chr>  <chr>
#>  1 field_check         Passed Passed: required fields VARNAME, VARDESC, UNITS, ~
#>  2 pkg_field_check     Passed Passed: package-level required fields TYPE, MIN, ~
#>  3 dimension_check     Passed Passed: the variable count matches between the da~
#>  4 name_check          Passed Passed: the variable names match between the data~
#>  5 id_check            Passed Passed: All ID variable checks passed.
#>  6 row_check           Passed Passed: no blank or duplicate rows detected in da~
#>  7 NA_check            Passed Passed: no NA values detected in data set.
#>  8 type_check          Passed Passed: All TYPE entries found are accepted by db~
#>  9 values_check        Passed Passed: all four VALUES checks look good.
#> 10 integer_check       Passed Passed: all variables listed as TYPE integer appe~
#> 11 decimal_check       Passed Passed: all variables listed as TYPE decimal appe~
#> 12 misc_format_check   Passed Passed: no check-specific formatting issues ident~
#> 13 description_check   Failed ERROR: missing and duplicate descriptions found i~
#> 14 minmax_check        Passed Passed: when provided, all variables are within t~
#> 15 missing_value_check Failed ERROR: some variables have non-encoded missing va~
#> --------------------
#> description_check: Failed
#> ERROR: missing and duplicate descriptions found in data dictionary.
#> $description_check.Info
#> # A tibble: 4 x 2
#>   VARNAME  VARDESC
#>   <chr>    <chr>
#> 1 PREGNANT <NA>
#> 2 REACT    <NA>
#> 3 HEIGHT   Height of participant
#> 4 WEIGHT   Height of participant
#>
#> --------------------
#> missing_value_check: Failed
#> ERROR: some variables have non-encoded missing value codes.
```

```
#> $missing_value_check.Info
#>     VARNAME VALUE MEANING  PASS
#> 16 CUFFSIZE -9999    <NA> FALSE
#>
#> --------------------
```

As you can see, now 13 out of 15 checks pass, but the workflow fails at `description_check` and `missing_value_check`. Specifically, in `description_check` we see that variables `PREGNANT` and `REACT` were identified as having missing variable descriptions (`VARDESC`), and variables `HEIGHT` and `WEIGHT` incorrectly have identical descriptions. In `missing_value_check`, we see that the variable `CUFFSIZE` contains a `-9999` encoded value that is not specified in a `VALUES` column. While we have included several functions that support "quick fixes" (`add_missing_fields`, `name_correct`, `reorder_dictionary`, `reorder_data`, `id_first_data`, and `id_first_dict`), the issues identified here are a bit more complex and study-specific, so would need to be corrected manually in your data dictionary before moving on. For now, we will leave this example and move on to the next one!

6.1.2 Example 2

```
data(ExampleL)
```

```
e2_report <- check_report(DD.dict.L, DS.data.L)
#> # A tibble: 15 x 3
#>    Function            Status        Message
#>    <chr>               <chr>         <chr>
#>  1 field_check         Passed        Passed: required fields VARNAME, VARDESC, ~
#>  2 pkg_field_check     Passed        Passed: package-level required fields TYPE~
#>  3 dimension_check     Passed        Passed: the variable count matches between~
#>  4 name_check          Failed        ERROR: the variable names DO NOT match bet~
#>  5 id_check            Passed        Passed: All ID variable checks passed.
#>  6 row_check           Passed        Passed: no blank or duplicate rows detecte~
#>  7 NA_check            Not attempted ERROR: Required pre-check name_check faile~
#>  8 type_check          Passed        Passed: All TYPE entries found are accepte~
#>  9 values_check        Failed        ERROR: at least one VALUES check flagged p~
#> 10 integer_check       Not attempted ERROR: Required pre-check name_check faile~
#> 11 decimal_check       Not attempted ERROR: Required pre-check name_check faile~
#> 12 misc_format_check   Failed        ERROR: at least one check failed.
#> 13 description_check   Failed        ERROR: missing and duplicate descriptions ~
#> 14 minmax_check        Not attempted ERROR: Required pre-check name_check faile~
#> 15 missing_value_check Not attempted ERROR: Required pre-check name_check faile~
#> --------------------
#> name_check: Failed
#> ERROR: the variable names DO NOT match between the data dictionary and the data. If
↪  the intention behind the variable names is correct, consider using the name_correct
↪  function to automatically rename variables to match.
#> $name_check.Info
#> # A tibble: 2 x 2
#>   Data                Dict
#>   <chr>               <chr>
#> 1 Data: SMOKING_HX    Dict: SMOKING_HISTORY
#> 2 Data: HX_DEPRESSION Dict: DEPRESSION_HX
```

```
#>
#> --------------------
#> values_check: Failed
#> ERROR: at least one VALUES check flagged potentials issues. See Information for more
↪  details.
#> $values_check.Info
#>    column_name values.check            vname                   type
#> 4      VALUES3        FALSE         CUFFSIZE integer, encoded value
#> 6       VALUES        FALSE              HTN integer, encoded value
#> 7       VALUES        FALSE PERCEIVED_HEALTH integer, encoded value
#> 9      VALUES5        FALSE               28 integer, encoded value
#> 10     VALUES4        FALSE               28 integer, encoded value
#> 12     VALUES2        FALSE               16 integer, encoded value
#> 14      VALUES        FALSE           RESIST integer, encoded value
#> 15      VALUES        FALSE        SAMPLE_ID                integer
#> 16      VALUES        FALSE              SEX                integer
#>                                                  problematic_description
#> 4                                                          2 means large
#> 6                                                          0 indicates no
#> 7  Between 1 and 10 with higher values indicating better perceived health
#> 9                                                          5 = a great deal
#> 10                                                         4 = quite a bit
#> 12                                                               1 =medium
#> 14                                                                    <NA>
#> 15                                                    -9999=missing value
#> 16                                                                  0=male
#>                                                                         check
#> 4                  Check 1: Is an equals sign present for all values columns?
#> 6                  Check 1: Is an equals sign present for all values columns?
#> 7                  Check 1: Is an equals sign present for all values columns?
#> 9  Check 2: Are there any leading/trailing spaces near the first equals sign?
#> 10 Check 2: Are there any leading/trailing spaces near the first equals sign?
#> 12 Check 2: Are there any leading/trailing spaces near the first equals sign?
#> 14  Check 3: Do all variables of TYPE encoded have at least one VALUES entry?
#> 15            Check 4: Are all variables with VALUES entries of TYPE encoded?
#> 16            Check 4: Are all variables with VALUES entries of TYPE encoded?
#>
#> --------------------
#> misc_format_check: Failed
#> ERROR: at least one check failed.
#> $misc_formatting_check.Info
#> # A tibble: 9 x 6
#>   check.name check.description                   check~1 details col.n~2 correct
#>   <chr>      <chr>                               <chr>   <lgl>   <chr>   <lgl>
#> 1 Check 1    Empty variable name check           Passed  NA      <NA>    NA
#> 2 Check 2    Duplicate variable name check       Passed  NA      <NA>    NA
#> 3 Check 3    Check for use of `dbgap` in variab~ Passed  NA      <NA>    NA
#> 4 Check 4    Duplicate dictionary column name c~ Passed  NA      <NA>    NA
#> 5 Check 5    Column names after `VALUES` should~ Failed  NA      VALUES2 FALSE
#> 6 Check 5    Column names after `VALUES` should~ Failed  NA      VALUES3 FALSE
#> 7 Check 5    Column names after `VALUES` should~ Failed  NA      VALUES4 FALSE
#> 8 Check 5    Column names after `VALUES` should~ Failed  NA      VALUES5 FALSE
#> 9 Check 5    Column names after `VALUES` should~ Failed  NA      VALUES6 FALSE
```

```
#> # ... with abbreviated variable names 1: check.status, 2: col.name
#>
#> --------------------
#> description_check: Failed
#> ERROR: missing and duplicate descriptions found in data dictionary.
#> $description_check.Info
#> # A tibble: 2 x 2
#>   VARNAME  VARDESC
#>   <chr>    <chr>
#> 1 PREGNANT <NA>
#> 2 REACT    <NA>
#>
#> --------------------
```

In example 2, we see that the first three checks (`field_check`, `pkg_field_check`, and `dimension_check`) and several others further down the workflow pass, but the fourth check (`name_check`) fails. Looking at the `check_report` output more closely, we see that there are two variables with names that do not match between the data dictionary and data set.

Before we move on to investigate this issue further, please note that we could arrive at the same conclusion using the functions individually (rather than the complete workflow approach implemented in `check_report`):

```
field_check(DD.dict.L) # pass
#> $Message
#> [1] "Passed: required fields VARNAME, VARDESC, UNITS, and VALUES present in the data
↪  dictionary."
pkg_field_check(DD.dict.L) # pass
#> $Message
#> [1] "Passed: package-level required fields TYPE, MIN, and MAX present in the data
↪  dictionary."
dimension_check(DD.dict.L, DS.data.L) # pass
#> $Message
#> [1] "Passed: the variable count matches between the data dictionary and the data."
#>
#> $Information
#> Variables in dictionary       Variables in data
#>                      30                      30
name_check(DD.dict.L, DS.data.L) # failed
#> $Message
#> [1] "ERROR: the variable names DO NOT match between the data dictionary and the data.
↪  If the intention behind the variable names is correct, consider using the
↪  name_correct function to automatically rename variables to match."
#>
#> $Information
#> # A tibble: 2 x 2
#>   Data                Dict
#>   <chr>               <chr>
#> 1 Data: SMOKING_HX    Dict: SMOKING_HISTORY
#> 2 Data: HX_DEPRESSION Dict: DEPRESSION_HX
```

In looking more closely at the `name_check` output, we then see that, while the "intent" between the names match (i.e., "hx" is sometimes used as shorthand for "history"), there are a couple of discrepancies between

the data dictionary and data set. Luckily, we have included a "quick fix" for this simple issue as implemented in the `name_correct` function so that you can continue working through the checks. Specifically, `name_correct` updates the names in the data set to match the names listed in the data dictionary. Similarly, if the variable names in the data dictionary and data set matched identically, but were in the wrong order, the `reorder_dictionary` function could be used to create a new version of the data dictionary to match the order presented in the data set (see Example 5)! Back to the example at hand, though – let's give the `name_correct` function a try now!

```
DS.data_updated <- name_correct(DD.dict.L, DS.data.L)
#> $Message
#> [1] "CORRECTED ERROR: the variable names differ between the data dictionary and the
↪  data. **ALERT** Renaming variable(s) to match those listed in the data dictionary."
#>
#> $Information
#> # A tibble: 2 x 3
#>   Data                              Dict                              New.Data
#>   <chr>                             <chr>                             <chr>
#> 1 Original data name: SMOKING_HX    Dictionary name: SMOKING_HISTORY New data n~
#> 2 Original data name: HX_DEPRESSION Dictionary name: DEPRESSION_HX   New data n~
```

Now that our error has been corrected, let's return to `check_report`. Similar to above, be sure to call in our updated data set!

```
# Calling in updated data set
e2_report.v2 <- check_report(DD.dict.L, DS.data_updated,
                non.NA.missing.codes=c(-4444, -9999))
#> Warning: Expected 2 pieces. Missing pieces filled with `NA` in 1 rows [3].
#> Warning: Expected 2 pieces. Missing pieces filled with `NA` in 1 rows [1].
#> Expected 2 pieces. Missing pieces filled with `NA` in 1 rows [1].
#> # A tibble: 15 x 3
#>    Function            Status Message
#>    <chr>               <chr>  <chr>
#>  1 field_check         Passed Passed: required fields VARNAME, VARDESC, UNITS, ~
#>  2 pkg_field_check     Passed Passed: package-level required fields TYPE, MIN, ~
#>  3 dimension_check     Passed Passed: the variable count matches between the da~
#>  4 name_check          Passed Passed: the variable names match between the data~
#>  5 id_check            Passed Passed: All ID variable checks passed.
#>  6 row_check           Passed Passed: no blank or duplicate rows detected in da~
#>  7 NA_check            Passed Passed: no NA values detected in data set.
#>  8 type_check          Passed Passed: All TYPE entries found are accepted by db~
#>  9 values_check        Failed ERROR: at least one VALUES check flagged potentia~
#> 10 integer_check       Failed ERROR: some variables listed as TYPE integer do n~
#> 11 decimal_check       Failed ERROR: some variables listed as TYPE decimal do n~
#> 12 misc_format_check   Failed ERROR: at least one check failed.
#> 13 description_check   Failed ERROR: missing and duplicate descriptions found i~
#> 14 minmax_check        Failed ERROR: some variables have values outside of the ~
#> 15 missing_value_check Failed ERROR: some variables have non-encoded missing va~
#> --------------------
#> values_check: Failed
#> ERROR: at least one VALUES check flagged potentials issues. See Information for more
↪  details.
#> $values_check.Info
#>    column_name values.check            vname                     type
```

```
#> 4      VALUES3        FALSE         CUFFSIZE integer, encoded value
#> 6       VALUES        FALSE              HTN integer, encoded value
#> 7       VALUES        FALSE PERCEIVED_HEALTH integer, encoded value
#> 9      VALUES5        FALSE               28 integer, encoded value
#> 10     VALUES4        FALSE               28 integer, encoded value
#> 12     VALUES2        FALSE               16 integer, encoded value
#> 14      VALUES        FALSE           RESIST integer, encoded value
#> 15      VALUES        FALSE        SAMPLE_ID                integer
#> 16      VALUES        FALSE              SEX                integer
#>                                                     problematic_description
#> 4                                                               2 means large
#> 6                                                              0 indicates no
#> 7  Between 1 and 10 with higher values indicating better perceived health
#> 9                                                            5 = a great deal
#> 10                                                           4 = quite a bit
#> 12                                                                 1 =medium
#> 14                                                                      <NA>
#> 15                                                       -9999=missing value
#> 16                                                                    0=male
#>                                                                         check
#> 4                  Check 1: Is an equals sign present for all values columns?
#> 6                  Check 1: Is an equals sign present for all values columns?
#> 7                  Check 1: Is an equals sign present for all values columns?
#> 9  Check 2: Are there any leading/trailing spaces near the first equals sign?
#> 10 Check 2: Are there any leading/trailing spaces near the first equals sign?
#> 12 Check 2: Are there any leading/trailing spaces near the first equals sign?
#> 14  Check 3: Do all variables of TYPE encoded have at least one VALUES entry?
#> 15            Check 4: Are all variables with VALUES entries of TYPE encoded?
#> 16            Check 4: Are all variables with VALUES entries of TYPE encoded?
#>
#> --------------------
#> integer_check: Failed
#> ERROR: some variables listed as TYPE integer do not appear to be integers.
#> $integer_check.Info
#> [1] "BP_DIASTOLIC"    "SMOKING_HISTORY"
#>
#> --------------------
#> decimal_check: Failed
#> ERROR: some variables listed as TYPE decimal do not appear to be decimals.
#> $decimal_check.Info
#> [1] "ABD_SKF" "SUP_SKF"
#>
#> --------------------
#> misc_format_check: Failed
#> ERROR: at least one check failed.
#> $misc_formatting_check.Info
#> # A tibble: 9 x 6
#>   check.name check.description                   check~1 details col.n~2 correct
#>   <chr>      <chr>                               <chr>   <lgl>   <chr>   <lgl>
#> 1 Check 1    Empty variable name check           Passed  NA      <NA>    NA
#> 2 Check 2    Duplicate variable name check       Passed  NA      <NA>    NA
#> 3 Check 3    Check for use of `dbgap` in variab~ Passed  NA      <NA>    NA
#> 4 Check 4    Duplicate dictionary column name c~ Passed  NA      <NA>    NA
```

```
#> 5 Check 5    Column names after `VALUES` should~ Failed  NA      VALUES2 FALSE
#> 6 Check 5    Column names after `VALUES` should~ Failed  NA      VALUES3 FALSE
#> 7 Check 5    Column names after `VALUES` should~ Failed  NA      VALUES4 FALSE
#> 8 Check 5    Column names after `VALUES` should~ Failed  NA      VALUES5 FALSE
#> 9 Check 5    Column names after `VALUES` should~ Failed  NA      VALUES6 FALSE
#> # ... with abbreviated variable names 1: check.status, 2: col.name
#>
#> --------------------
#> description_check: Failed
#> ERROR: missing and duplicate descriptions found in data dictionary.
#> $description_check.Info
#> # A tibble: 2 x 2
#>   VARNAME  VARDESC
#>   <chr>    <chr>
#> 1 PREGNANT <NA>
#> 2 REACT    <NA>
#>
#> --------------------
#> minmax_check: Failed
#> ERROR: some variables have values outside of the MIN to MAX range.
#> $minmax_check.Info
#> # A tibble: 1 x 5
#>   Trait              Check ListedMin ListedMax OutOfRangeValues
#>   <chr>              <lgl>     <dbl>     <dbl> <list>
#> 1 PERCEIVED_CONFLICT FALSE         1        15 <int [11]>
#>
#> --------------------
#> missing_value_check: Failed
#> ERROR: some variables have non-encoded missing value codes.
#> $missing_value_check.Info
#>     VARNAME VALUE MEANING  PASS
#> 14   RESIST -9999    <NA> FALSE
#> 16 CUFFSIZE -9999    <NA> FALSE
#>
#> --------------------
```

We now see that `name_check` now passes, along with several other functions in the workflow, but we have failed on `values_check` and several others.

Investigating this check failure further by looking at the `check_report` output, we see a few issues that, due to the subjectivity and complexity of different data set, will need to be manually corrected before moving on. For the purposes of this tutorial, we will now leave this data set to move on to a new one, but in reality, we would correct this issue and return again to `check_report`.

6.1.3 Example 3

```
data(ExampleB)
```

Again, we will start with the `check_report` function.

```
e3_report <- check_report(DD.dict.B, DS.data.B)
#> # A tibble: 15 x 3
#>    Function            Status Message
#>    <chr>               <chr>  <chr>
#>  1 field_check         Passed Passed: required fields VARNAME, VARDESC, UNITS, ~
#>  2 pkg_field_check     Passed Passed: package-level required fields TYPE, MIN, ~
#>  3 dimension_check     Passed Passed: the variable count matches between the da~
#>  4 name_check          Passed Passed: the variable names match between the data~
#>  5 id_check            Passed Passed: All ID variable checks passed.
#>  6 row_check           Passed Passed: no blank or duplicate rows detected in da~
#>  7 NA_check            Passed Passed: no NA values detected in data set.
#>  8 type_check          Passed Passed: All TYPE entries found are accepted by db~
#>  9 values_check        Passed Passed: all four VALUES checks look good.
#> 10 integer_check       Passed Passed: all variables listed as TYPE integer appe~
#> 11 decimal_check       Passed Passed: all variables listed as TYPE decimal appe~
#> 12 misc_format_check   Passed Passed: no check-specific formatting issues ident~
#> 13 description_check   Passed Passed: unique description present for all variab~
#> 14 minmax_check        Passed Passed: when provided, all variables are within t~
#> 15 missing_value_check Passed Passed: all missing value codes have a correspond~
#> [1] "All 15 checks passed."
```

In the above chunk, `check_report` determines that all 15 checks were passed! But ALERT — this is misleading as we forgot to include an important parameter!!!!! Rerunning the check with our missing value codes defined, we now see an issue at `missing_value_check`, which underscores the importance of specifying missing value codes.

```
e3_report.v2 <- check_report(DD.dict.B, DS.data.B, non.NA.missing.codes=c(-9999))
#> # A tibble: 15 x 3
#>    Function            Status Message
#>    <chr>               <chr>  <chr>
#>  1 field_check         Passed Passed: required fields VARNAME, VARDESC, UNITS, ~
#>  2 pkg_field_check     Passed Passed: package-level required fields TYPE, MIN, ~
#>  3 dimension_check     Passed Passed: the variable count matches between the da~
#>  4 name_check          Passed Passed: the variable names match between the data~
#>  5 id_check            Passed Passed: All ID variable checks passed.
#>  6 row_check           Passed Passed: no blank or duplicate rows detected in da~
#>  7 NA_check            Passed Passed: no NA values detected in data set.
#>  8 type_check          Passed Passed: All TYPE entries found are accepted by db~
#>  9 values_check        Passed Passed: all four VALUES checks look good.
#> 10 integer_check       Passed Passed: all variables listed as TYPE integer appe~
#> 11 decimal_check       Passed Passed: all variables listed as TYPE decimal appe~
#> 12 misc_format_check   Passed Passed: no check-specific formatting issues ident~
#> 13 description_check   Passed Passed: unique description present for all variab~
#> 14 minmax_check        Passed Passed: when provided, all variables are within t~
#> 15 missing_value_check Failed ERROR: some variables have non-encoded missing va~
#> --------------------
#> missing_value_check: Failed
#> ERROR: some variables have non-encoded missing value codes.
#> $missing_value_check.Info
#>     VARNAME VALUE MEANING  PASS
#> 13 CUFFSIZE -9999    <NA> FALSE
#>
#> --------------------
```

If you are not immediately sure what your missing value codes are, you can use our `value_meaning_table` utility/awareness function.

```
value_meaning_table(DD.dict.B)
#>                 VARNAME                      TYPE VALUE                                  MEANING
#> 2             SAMPLE_ID integer, encoded value -9999                            missing value
#> 3                   SEX integer, encoded value     0                                     male
#> 4                   SEX integer, encoded value     1                                   female
#> 5                HEIGHT decimal, encoded value -9999                            missing value
#> 6                WEIGHT decimal, encoded value -9999                            missing value
#> 7                   BMI decimal, encoded value -9999                            missing value
#> 8               OBESITY integer, encoded value     0                                       no
#> 9               OBESITY integer, encoded value     1                                      yes
#> 10              OBESITY integer, encoded value -9999                            missing value
#> 11             ABD_CIRC decimal, encoded value -9999                            missing value
#> 12             HIP_CIRC decimal, encoded value -9999                            missing value
#> 13              ABD_SKF integer, encoded value -9999                            missing value
#> 14              SUP_SKF integer, encoded value -9999                            missing value
#> 15               RESIST integer, encoded value -9999                            missing value
#> 16                REACT integer, encoded value -9999                            missing value
#> 17             CUFFSIZE integer, encoded value     0                                    small
#> 18             CUFFSIZE integer, encoded value     1                                   medium
#> 19             CUFFSIZE integer, encoded value     2                                    large
#> 20             CUFFSIZE integer, encoded value     3                              extra large
#> 21          BP_SYSTOLIC integer, encoded value -9999                            missing value
#> 22         BP_DIASTOLIC integer, encoded value -9999                            missing value
#> 23                  HTN integer, encoded value     0                                       no
#> 24                  HTN integer, encoded value     1                                      yes
#> 25                  HTN integer, encoded value -9999                            missing value
#> 26           SMOKING_HX integer, encoded value     0                                       no
#> 27           SMOKING_HX integer, encoded value     1                                      yes
#> 28           SMOKING_HX integer, encoded value -9999                            missing value
#> 29 LENGTH_SMOKING_YEARS decimal, encoded value -9999                            missing value
#> 30 LENGTH_SMOKING_YEARS decimal, encoded value -4444 not applicable, no history of smoking
#> 31           HEART_RATE integer, encoded value -9999                            missing value
#> 32    PHYSICAL_ACTIVITY integer, encoded value -9999                            missing value
#> 33                HX_DM integer, encoded value     0                                       no
#> 34                HX_DM integer, encoded value     1                                      yes
#> 35                HX_DM integer, encoded value -9999                            missing value
#> 36            HX_STROKE integer, encoded value     0                                       no
```

```
#> 37            HX_STROKE integer, encoded value     1                                       yes
#> 38            HX_STROKE integer, encoded value -9999                             missing value
#> 39           HX_ANXIETY integer, encoded value     0                                        no
#> 40           HX_ANXIETY integer, encoded value     1                                       yes
#> 41           HX_ANXIETY integer, encoded value -9999                             missing value
#> 42        HX_DEPRESSION integer, encoded value     0                                        no
#> 43        HX_DEPRESSION integer, encoded value     1                                       yes
#> 44        HX_DEPRESSION integer, encoded value -9999                             missing value
#> 45       SOCIAL_SUPPORT integer, encoded value     1                               very little
#> 46       SOCIAL_SUPPORT integer, encoded value     2                                    little
#> 47       SOCIAL_SUPPORT integer, encoded value     3                                  moderate
#> 48       SOCIAL_SUPPORT integer, encoded value     4                               quite a bit
#> 49       SOCIAL_SUPPORT integer, encoded value     5                              a great deal
#> 50   PERCEIVED_CONFLICT integer, encoded value     1        lowest possible social conflict
#> 51   PERCEIVED_CONFLICT integer, encoded value    30       highest possible social conflict
#> 52     PERCEIVED_HEALTH integer, encoded value     1     poorest possible perceived health
#> 53     PERCEIVED_HEALTH integer, encoded value    10        best possible perceived health
```

So here we see that -9999 is a verified missing value code in this example.

6.1.4 Example 4

```
data(ExampleH)
```

```
e4_report <- check_report(DD.dict.H, DS.data.H, non.NA.missing.codes=c(-4444, -9999))
#> # A tibble: 15 x 3
#>    Function            Status Message
#>    <chr>               <chr>  <chr>
#>  1 field_check         Passed Passed: required fields VARNAME, VARDESC, UNITS, ~
#>  2 pkg_field_check     Passed Passed: package-level required fields TYPE, MIN, ~
#>  3 dimension_check     Passed Passed: the variable count matches between the da~
#>  4 name_check          Passed Passed: the variable names match between the data~
#>  5 id_check            Passed Passed: All ID variable checks passed.
#>  6 row_check           Passed Passed: no blank or duplicate rows detected in da~
#>  7 NA_check            Passed Passed: no NA values detected in data set.
#>  8 type_check          Passed Passed: All TYPE entries found are accepted by db~
#>  9 values_check        Passed Passed: all four VALUES checks look good.
#> 10 integer_check       Failed ERROR: some variables listed as TYPE integer do n~
#> 11 decimal_check       Passed Passed: all variables listed as TYPE decimal appe~
#> 12 misc_format_check   Passed Passed: no check-specific formatting issues ident~
#> 13 description_check   Passed Passed: unique description present for all variab~
#> 14 minmax_check        Passed Passed: when provided, all variables are within t~
#> 15 missing_value_check Passed Passed: all missing value codes have a correspond~
#> --------------------
#> integer_check: Failed
#> ERROR: some variables listed as TYPE integer do not appear to be integers.
#> $integer_check.Info
#> [1] "SUP_SKF"
#>
#> --------------------
```

Note that in this example, we see an error at `integer_check`. Let's investigate this further.

Specifically, we can use the awareness function to grep (i.e., search) for this variable name in the dictionary.

```
dictionary_search(DD.dict.H, search.term=c("SUP_SKF"), search.column=c("VARNAME"))
#> # A tibble: 1 x 7
#>   VARNAME VARDESC                       UNITS TYPE               MIN   MAX VALUES
#>   <chr>   <chr>                         <chr> <chr>            <dbl> <dbl> <chr>
#> 1 SUP_SKF Suprailiac skinfold thickness mm    integer, encod~     NA    NA -9999~
```

We can also look at the values in the data set to see that, in fact, there are some values that are decimals (not integers as the dictionary suggests).

```
table(DS.data.H$SUP_SKF)
#>
#>  -9999     12     22 23.888     24     25     26     27     28 28.254     29
#>      3      2      4      1      4     11      4      6      2      1      1
#>     34     35     36     37     38     39  39.12     40     42     44     45
#>      4      7      3      2      3      6      1      9      2      6      3
#>     46     48     51     52
#>      4      3      2      6
```

We can also use this awareness function to grep any variables that are described as "skinfold" measurements to evaluate data TYPE across variables.

```
dictionary_search(DD.dict.H, search.term=c("skinfold"))
#> # A tibble: 2 x 7
#>   VARNAME VARDESC                        UNITS TYPE              MIN   MAX VALUES
#>   <chr>   <chr>                          <chr> <chr>           <dbl> <dbl> <chr>
#> 1 ABD_SKF Abdominal skinfold thickness  mm    integer, encod~    NA    NA -9999~
#> 2 SUP_SKF Suprailiac skinfold thickness mm    integer, encod~    NA    NA -9999~
```

Above we see that both abdominal and suprailiac skinfold thickness are listed as integers in the data dictionary, and thought to have been measured to the nearest mm.

```
table(DS.data.H$ABD_SKF)
#>
#> -9999    14    18    21    22    23    24    25    26    27    28    29    30
#>     3     1     2     2     1     4    11    22     4     1     6     4     2
#>    31    32    34    35    36    38    39    40    41    42    45    51    54
#>     3     2     5     4     5     6     2     1     1     1     1     1     1
#>    61    65    68
#>     2     1     1
```

While ABD_SKF appears to be a true integer, SUP_SKF appears to have some decimal places. This error could be corrected either by listing SUP_SKF as TYPE decimal, or by investigating if the data set has a recording/measurement error.

6.1.5 Example 5

```
data(ExampleN)
```

```
e5_report <- check_report(DD.dict.N, DS.data.N)
#> # A tibble: 15 x 3
#>    Function            Status        Message
#>    <chr>               <chr>         <chr>
#>  1 field_check         Passed        Passed: required fields VARNAME, VARDESC, ~
#>  2 pkg_field_check     Passed        Passed: package-level required fields TYPE~
#>  3 dimension_check     Passed        Passed: the variable count matches between~
#>  4 name_check          Failed        ERROR: the variable names match between th~
#>  5 id_check            Passed        Passed: All ID variable checks passed.
#>  6 row_check           Passed        Passed: no blank or duplicate rows detecte~
#>  7 NA_check            Not attempted ERROR: Required pre-check name_check faile~
#>  8 type_check          Passed        Passed: All TYPE entries found are accepte~
#>  9 values_check        Failed        ERROR: at least one VALUES check flagged p~
#> 10 integer_check       Not attempted ERROR: Required pre-check name_check faile~
#> 11 decimal_check       Not attempted ERROR: Required pre-check name_check faile~
#> 12 misc_format_check   Failed        ERROR: at least one check failed.
#> 13 description_check   Failed        ERROR: missing and duplicate descriptions ~
#> 14 minmax_check        Not attempted ERROR: Required pre-check name_check faile~
#> 15 missing_value_check Not attempted ERROR: Required pre-check name_check faile~
#> --------------------
```

```
#> name_check: Failed
#> ERROR: the variable names match between the data dictionary and the data, but they are
↪  in the wrong order. Consider using reorder_dictionary function to automatically
↪  reorder the dictionary so that you can continue working through the checks.
#> $name_check.Info
#> # A tibble: 10 x 2
#>    Data                         Dict
#>    <chr>                        <chr>
#>  1 Data: ABD_CIRC               Dict: HIP_CIRC
#>  2 Data: HIP_CIRC               Dict: ABD_SKF
#>  3 Data: ABD_SKF                Dict: SUP_SKF
#>  4 Data: SUP_SKF                Dict: ABD_CIRC
#>  5 Data: BP_DIASTOLIC           Dict: HTN
#>  6 Data: HTN                    Dict: SMOKING_HX
#>  7 Data: SMOKING_HX             Dict: LENGTH_SMOKING_YEARS
#>  8 Data: LENGTH_SMOKING_YEARS Dict: HEART_RATE
#>  9 Data: HEART_RATE             Dict: PHYSICAL_ACTIVITY
#> 10 Data: PHYSICAL_ACTIVITY      Dict: BP_DIASTOLIC
#>
#> --------------------
#> values_check: Failed
#> ERROR: at least one VALUES check flagged potentials issues. See Information for more
↪  details.
#> $values_check.Info
#>    column_name values.check            vname                    type
#> 4      VALUES3        FALSE         CUFFSIZE integer, encoded value
#> 6       VALUES        FALSE              HTN integer, encoded value
#> 7       VALUES        FALSE PERCEIVED_HEALTH integer, encoded value
#> 9      VALUES5        FALSE               28 integer, encoded value
#> 10     VALUES4        FALSE               28 integer, encoded value
#> 12     VALUES2        FALSE               16 integer, encoded value
#> 14      VALUES        FALSE           RESIST integer, encoded value
#> 15      VALUES        FALSE        SAMPLE_ID                integer
#> 16      VALUES        FALSE              SEX                integer
#>                                                     problematic_description
#> 4                                                             2 means large
#> 6                                                             0 indicates no
#> 7  Between 1 and 10 with higher values indicating better perceived health
#> 9                                                           5 = a great deal
#> 10                                                           4 = quite a bit
#> 12                                                                 1 =medium
#> 14                                                                      <NA>
#> 15                                                        -9999=missing value
#> 16                                                                    0=male
#>                                                                        check
#> 4                  Check 1: Is an equals sign present for all values columns?
#> 6                  Check 1: Is an equals sign present for all values columns?
#> 7                  Check 1: Is an equals sign present for all values columns?
#> 9  Check 2: Are there any leading/trailing spaces near the first equals sign?
#> 10 Check 2: Are there any leading/trailing spaces near the first equals sign?
#> 12 Check 2: Are there any leading/trailing spaces near the first equals sign?
#> 14  Check 3: Do all variables of TYPE encoded have at least one VALUES entry?
#> 15            Check 4: Are all variables with VALUES entries of TYPE encoded?
```

```
#> 16            Check 4: Are all variables with VALUES entries of TYPE encoded?
#>
#> --------------------
#> misc_format_check: Failed
#> ERROR: at least one check failed.
#> $misc_formatting_check.Info
#> # A tibble: 9 x 6
#>   check.name check.description                   check~1 details col.n~2 correct
#>   <chr>      <chr>                               <chr>   <lgl>   <chr>   <lgl>
#> 1 Check 1    Empty variable name check           Passed  NA      <NA>    NA
#> 2 Check 2    Duplicate variable name check       Passed  NA      <NA>    NA
#> 3 Check 3    Check for use of `dbgap` in variab~ Passed  NA      <NA>    NA
#> 4 Check 4    Duplicate dictionary column name c~ Passed  NA      <NA>    NA
#> 5 Check 5    Column names after `VALUES` should~ Failed  NA      VALUES2 FALSE
#> 6 Check 5    Column names after `VALUES` should~ Failed  NA      VALUES3 FALSE
#> 7 Check 5    Column names after `VALUES` should~ Failed  NA      VALUES4 FALSE
#> 8 Check 5    Column names after `VALUES` should~ Failed  NA      VALUES5 FALSE
#> 9 Check 5    Column names after `VALUES` should~ Failed  NA      VALUES6 FALSE
#> # ... with abbreviated variable names 1: check.status, 2: col.name
#>
#> --------------------
#> description_check: Failed
#> ERROR: missing and duplicate descriptions found in data dictionary.
#> $description_check.Info
#> # A tibble: 2 x 2
#>   VARNAME  VARDESC
#>   <chr>    <chr>
#> 1 PREGNANT <NA>
#> 2 REACT    <NA>
#>
#> --------------------
values_check(DD.dict.N)
#> $Message
#> [1] "ERROR: at least one VALUES check flagged potentials issues. See Information for
↪  more details."
#>
#> $Information
#>    column_name values.check            vname                    type
#> 4      VALUES3        FALSE         CUFFSIZE integer, encoded value
#> 6       VALUES        FALSE              HTN integer, encoded value
#> 7       VALUES        FALSE PERCEIVED_HEALTH integer, encoded value
#> 9      VALUES5        FALSE               28 integer, encoded value
#> 10     VALUES4        FALSE               28 integer, encoded value
#> 12     VALUES2        FALSE               16 integer, encoded value
#> 14      VALUES        FALSE           RESIST integer, encoded value
#> 15      VALUES        FALSE        SAMPLE_ID                 integer
#> 16      VALUES        FALSE              SEX                 integer
#>                                                   problematic_description
#> 4                                                            2 means large
#> 6                                                           0 indicates no
#> 7  Between 1 and 10 with higher values indicating better perceived health
#> 9                                                         5 = a great deal
#> 10                                                         4 = quite a bit
```

```
#> 12                                                                     1 =medium
#> 14                                                                          <NA>
#> 15                                                          -9999=missing value
#> 16                                                                        0=male
#>                                                                              check
#> 4                    Check 1: Is an equals sign present for all values columns?
#> 6                    Check 1: Is an equals sign present for all values columns?
#> 7                    Check 1: Is an equals sign present for all values columns?
#> 9  Check 2: Are there any leading/trailing spaces near the first equals sign?
#> 10 Check 2: Are there any leading/trailing spaces near the first equals sign?
#> 12 Check 2: Are there any leading/trailing spaces near the first equals sign?
#> 14  Check 3: Do all variables of TYPE encoded have at least one VALUES entry?
#> 15             Check 4: Are all variables with VALUES entries of TYPE encoded?
#> 16             Check 4: Are all variables with VALUES entries of TYPE encoded?
```

In this example, dbGaPCheckup informs us several issues — let's focus first on the `name_check` results. While the variable names match between the data dictionary and the data (in contrast to Example 2), they are now in the wrong order. Instead of fixing this issue manually outside of R, we can simply call the `reoder_dictionary` function as a "quick fix" and run the `name_check` function to confirm our update works!

```
DD.dict_updated <- reorder_dictionary(DD.dict.N, DS.data.N)
#> $Message
#> [1] "CORRECTED ERROR: the variable names match between the data dictionary and the
↪  data, but they were in the wrong order. ***ALERT**** this function has temporarily
↪  reordered the dictionary to match the data so that you can continue working through
↪  the checks."
#>
#> $Information
#> # A tibble: 10 x 3
#>    Data                        Dict                        New.Dict
#>    <chr>                       <chr>                       <chr>
#>  1 Data: ABD_CIRC              Dict: HIP_CIRC              Data: ABD_CIRC
#>  2 Data: HIP_CIRC              Dict: ABD_SKF               Data: HIP_CIRC
#>  3 Data: ABD_SKF               Dict: SUP_SKF               Data: ABD_SKF
#>  4 Data: SUP_SKF               Dict: ABD_CIRC              Data: SUP_SKF
#>  5 Data: BP_DIASTOLIC          Dict: HTN                   Data: BP_DIASTOLIC
#>  6 Data: HTN                   Dict: SMOKING_HX            Data: HTN
#>  7 Data: SMOKING_HX            Dict: LENGTH_SMOKING_YEARS Data: SMOKING_HX
#>  8 Data: LENGTH_SMOKING_YEARS Dict: HEART_RATE            Data: LENGTH_SMOKING_Y~
#>  9 Data: HEART_RATE            Dict: PHYSICAL_ACTIVITY    Data: HEART_RATE
#> 10 Data: PHYSICAL_ACTIVITY    Dict: BP_DIASTOLIC          Data: PHYSICAL_ACTIVITY
```

```
# Remember to call in the updated data dictionary!
name_check(DD.dict_updated, DS.data.N)
#> $Message
#> [1] "Passed: the variable names match between the data dictionary and the data."
#>
#> $Information
#> [1] "Variable names matched"
```

Above, we see that `name_check` now passes! Moving forward, we could simply return to our `check_report` workflow to search for other potential issues in finalizing our files for dbGaP submission.

6.1.6 Example 6

```
data(ExampleA)
```

As mentioned above, if you prefer, you can also simply run the individual checks that you are interested in rather than taking the complete workflow approach. Note that several package-specific pre-checks are embedded in many of the functions (e.g., `integer_check`).

```
id_check(DS.data.A)
#> $Message
#> [1] "Passed: All ID variable checks passed."
#>
#> $Information
#> # A tibble: 5 x 4
#>   check.name check.description                                 check.s~1 details
#>   <chr>      <chr>                                             <chr>     <chr>
#> 1 Check 1    Column 1 is labeled as 'SUBJECT_ID'.              Passed    The fi~
#> 2 Check 2    'SUBJECT_ID' is a column name in the data set.    Passed    'SUBJE~
#> 3 Check 3    'SUBJECT_ID' is a column name in the data set.    Passed    No ill~
#> 4 Check 4    No leading zeros detected in 'SUBJECT_ID' column. Passed    No lea~
#> 5 Check 5    No missing values for 'SUBJECT_ID'.               Passed    No mis~
#> # ... with abbreviated variable name 1: check.status
```

```
misc_format_check(DD.dict.A, DS.data.A)
#> $Message
#> [1] "Passed: no check-specific formatting issues identified."
#>
#> $Information
#> # A tibble: 5 x 4
#>   check.name check.description                        check.status details
#>   <chr>      <chr>                                    <chr>        <lgl>
#> 1 Check 1    Empty variable name check                Passed       NA
#> 2 Check 2    Duplicate variable name check            Passed       NA
#> 3 Check 3    Check for use of `dbgap` in variable names Passed       NA
#> 4 Check 4    Duplicate dictionary column name check   Passed       NA
#> 5 Check 5    Column names after `VALUES` should be empty Passed       NA
```

```
row_check(DD.dict.A, DS.data.A)
#> $Message
#> [1] "Passed: no blank or duplicate rows detected in data set or data dictionary."
```

```
NA_check(DD.dict.A, DS.data.A)
#> $Message
#> [1] "Passed: no NA values detected in data set."
```

```
minmax_check(DD.dict.A, DS.data.A)
#> $Message
#> [1] "ERROR: some variables have values outside of the MIN to MAX range."
#>
#> $Information
```

```
#> # A tibble: 1 x 5
#>   Trait    Check ListedMin ListedMax OutOfRangeValues
#>   <chr>    <lgl>     <dbl>     <dbl> <list>
#> 1 PREGNANT FALSE         0         1 <int [2]>
```

Above we see that an issue has been discovered at `minmax_check`. Let's investigate this further. The approach to view the "out of range values" is a bit cryptic, but it can be done with the following code.

```
b <- minmax_check(DD.dict.A, DS.data.A)
#> $Message
#> [1] "ERROR: some variables have values outside of the MIN to MAX range."
#>
#> $Information
#> # A tibble: 1 x 5
#>   Trait    Check ListedMin ListedMax OutOfRangeValues
#>   <chr>    <lgl>     <dbl>     <dbl> <list>
#> 1 PREGNANT FALSE         0         1 <int [2]>
b$Information[[1]]$OutOfRangeValues
#> [[1]]
#> [1] -4444 -9999
```

Here we see that we forgot to specify our missing value codes when we ran `minmax_check`, so they are being flagged as errors. Let's rerun the command specifying -4444 and -9999 as missing value codes.

```
minmax_check(DD.dict.A, DS.data.A, non.NA.missing.codes=c(-4444, -9999))
#> $Message
#> [1] "Passed: when provided, all variables are within the MIN to MAX range."
```

Now we see that our check passed for this data set!

6.2 Reporting functions

We have also created awareness and reporting functions that are not built into the complete workflow approach. These functions generate graphical and textual descriptions and awareness checks of the data in HTML format. These reports are designed to help you catch other potential errors in your data set. Note that the `create_report` generated is quite long however, so we recommend that you only submit subsets of variables at a time. Specification of missing value codes are also important for effective plotting. The commands are not ran here, as they work best when initiated interactively.

```
# Functions not run here as they work best when initiated interactively
# Awareness Report (See Appendix A for more details)
create_awareness_report(DD.dict, DS.data, non.NA.missing.codes=c(-9999, -4444),
   output.path= tempdir())

# Data Report (See Appendix B for more details)
create_report(DD.dict, DS.data, sex.split=TRUE, sex.name= "SEX",
   start = 3, end = 7, non.NA.missing.codes=c(-9999,-4444),
   output.path= tempdir(), open.html=TRUE)
```

For more details and to learn more, see the appendices below (`create_awareness_report`, Appendix A; `create_report`, Appendix B).

6.3 Label data function

Note that after your data dictionary is fully consistent with your data, you can use the `label_data` function to convert your data to labelled data, essentially embedding the data dictionary into the data for future use! This function uses Haven labelled data with SPSS style missing data codes to add non-missing information from the data dictionary as attributes to the data.

```
DS_labelled_data <- label_data(DD.dict.A, DS.data.A, non.NA.missing.codes=c(-9999))
labelled::var_label(DS_labelled_data$SEX)
#> [1] "Sex assigned at birth"
labelled::val_labels(DS_labelled_data$SEX)
#>   male female
#>      0      1
attributes(DS_labelled_data$SEX)
#> $labels
#>   male female
#>      0      1
#>
#> $label
#> [1] "Sex assigned at birth"
#>
#> $class
#> [1] "haven_labelled" "vctrs_vctr"     "integer"
#>
#> $TYPE
#> [1] "integer, encoded value"
#>
#> $MIN
#> [1] 0
#>
#> $MAX
#> [1] 1
labelled::na_values(DS_labelled_data$HX_DEPRESSION)
#> missing value
#>         -9999
```

7 Appendix: Reporting functions

As described above, there are a variety of awareness and reporting functions that are not built into the complete workflow approach. The purpose of this appendix is to highlight some of these features using the following example data.

```
data(ExampleB)
```

7.1 Appendix A: Awareness Report

Run `create_awareness_report`, which creates a nice .Rmd version of the below checks. While the output below is nearly identical to that you will see using the `create_awareness_report` function, for the purposes of this vignette, we have further expanded the annotation to assist in interpretation of the output through an example.

```
# Not run as works best when run interactively
create_awareness_report(DD.dict, DS.data, non.NA.missing.codes=c(-9999),
   output.path= tempdir())
```

7.1.1 Missingness Summary

This awareness function summarizes the amount of missingness in the data set.

```
missingness_summary(DS.data.B, non.NA.missing.codes = c(-9999), threshold = 95)
```

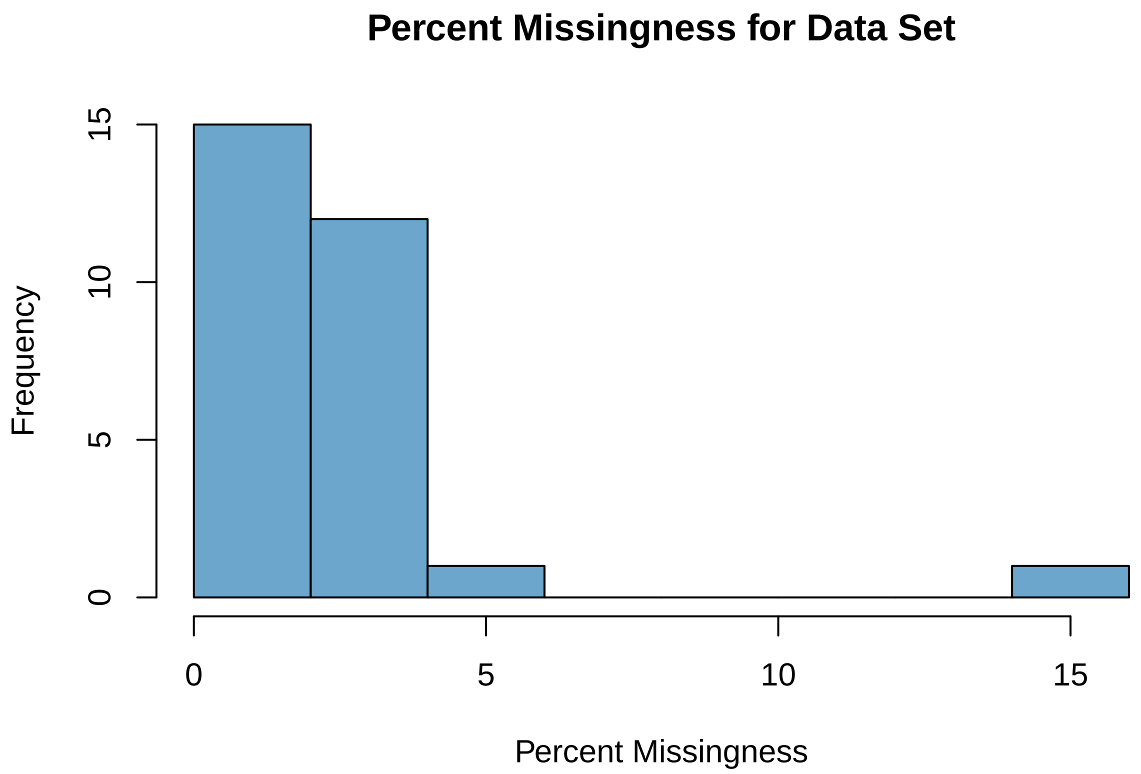


```
#> $Message
#> [1] "There are 0 variables with a percent missingness > 95% in your data set."
#>
#> $threshold_summary
#> [1] missing             percent_missingness
#> <0 rows> (or 0-length row.names)
#>
#> $full_missingness_summary
#>                      missing percent_missingness
#> SAMPLE_ID                 16                  16
#> SMOKING_HX                 5                   5
#> PHYSICAL_ACTIVITY          4                   4
#> WEIGHT                     3                   3
#> BMI                        3                   3
```

```
#> OBESITY                      3                   3
#> ABD_CIRC                     3                   3
#> HIP_CIRC                     3                   3
#> ABD_SKF                      3                   3
#> SUP_SKF                      3                   3
#> RESIST                       3                   3
#> REACT                        3                   3
#> HX_DM                        3                   3
#> HX_STROKE                    3                   3
#> HEIGHT                       2                   2
#> CUFFSIZE                     2                   2
#> BP_SYSTOLIC                  2                   2
#> BP_DIASTOLIC                 2                   2
#> HTN                          2                   2
#> HX_ANXIETY                   2                   2
#> HX_DEPRESSION                2                   2
#> SUBJECT_ID                   0                   0
#> AGE                          0                   0
#> SEX                          0                   0
#> LENGTH_SMOKING_YEARS         0                   0
#> HEART_RATE                   0                   0
#> SOCIAL_SUPPORT               0                   0
#> PERCEIVED_CONFLICT           0                   0
#> PERCEIVED_HEALTH             0                   0
```

Above we that there are 0 variables in our example data set that have a percent missingness >95%. Navigating through the output, we also see a complete summary of missingness in our data set, with `SAMPLE_ID` having the highest % missingness at 16%. Finally we see a histogram plotting missingness across our data set.

7.1.2 Values Missing Tables

In the `value_missing_table` function, for each variable, we have three sets of possible values:

(1) the set D of all the unique values observed in the data;

(2) the set V of all the values explicitly encoded in the VALUES columns of the data dictionary; and

(3) the set M of the missing value codes defined by the user via the `non.NA.missing.codes` argument.

This function examines various intersections of these three sets, providing awareness checks about possible issues of concern.

```
results.list <- value_missing_table(DD.dict.B, DS.data.B, non.NA.missing.codes =
↪  c(-9999))
#> $Message
#> [1] "Flag: at least one check flagged."
#>
#> $Information
#> # A tibble: 7 x 4
#>   check.name                          check.description                check~1 details
#>   <chr>                               <chr>                            <chr>   <named >
```

```
#> 1 Check A: In M, Not in D         "All missing value codes are ~ Flag    <tibble>
#> 2 Check B: In V, Not in D         "All value codes are in the d~ Flag    <tibble>
#> 3 Check C: In M, Not in V         "All missing value codes are ~ Flag    <tibble>
#> 4 Check D: In M & in D, not in V "All missing value codes are ~ Flag    <tibble>
#> 5 Check E: V NOT in M, NOT in D  "All value codes no defined a~ Passed  <chr>
#> 6 Awareness: NsetD vs. NsetV      "Size of Set D vs size of set~ Info    <tibble>
#> 7 Awareness: N_DnotM vs. N_VnotM "Size of Set D\\M vs size of ~ Info    <tibble>
#> # ... with abbreviated variable name 1: check.status
results <- results.list$report
```

7.1.2.1 Check A: If the user defines a missing value code that is not present in the data (In Set M and Not in Set D).

Table 5: Table Check A: List of variables for which user-defined missing value code is not present in the data.

VARNAME	AllMInD	NsetD	NsetM	NsetDAndSetM	MNotInD	MInD
SEX	FALSE	2	1	0	-9999	
LENGTH_SMOKING_YEARS	FALSE	12	1	0	-9999	
HEART_RATE	FALSE	44	1	0	-9999	
SOCIAL_SUPPORT	FALSE	5	1	0	-9999	
PERCEIVED_CONFLICT	FALSE	24	1	0	-9999	
PERCEIVED_HEALTH	FALSE	10	1	0	-9999	

The above table lists the variables for which the user-defined missing value code of `-9999` is not present in the data. These are not necessarily errors, however, as `dbGaPCheckup` reads `non.NA.missing.codes` as "global" missing value codes, even if a specific variable does not contain the code. For example, in the example data set, the SEX variable is complete, containing no missing value codes and only containing encoded values of 0=male, and 1=female, but `SEX` is flagged in the above variable list since it does not contain a `-9999` value. In other words, this variable's presence in the above list is NOT an issue that we should be concerned about. This function is intended only to bring awareness to potential errors in your data (e.g., perhaps you knew that the sex variable was missing for 5 participants for your specific study.)

Interpretation of table column names:
–> `AllMInD`: Variable-specific check result communicating if user-defined missing value code(s) are detected in the data set (FALSE=no).
–> `NsetD`: Number of values (or levels) detected in the data (e.g., in this example, `SEX` has two levels [0=male, 1=female]).
–> `NsetM`: Number of missing value codes defined (e.g., in this example, 1 user-defined missing value code [`-9999`] was defined).
–> `NsetDAndSetM`: Number of occurrences detected in both the data set and the user-defined missing value code (e.g., here 0 overlap for these variables, but if a second missing value code were defined, we might see a 1 here).
–> `MNotInD`: User-defined missing value code the function checked for (e.g., in this example, `-9999`).
–> `MInD`: Variable-specific number; user-defined missing value codes detected in the data (e.g., in this example, 0).

7.1.2.2 Check B: If a VALUES entry defines an encoded code value, but that value is not present in the data (In Set V and Not in Set D).

Table 6: Table Check B: List of variables for which a VALUES entry defines an encoded code value, but that value is not present in the data.

VARNAME	AllVsInD	NsetD	NsetV	NsetDAndSetV	VsNotInD
LENGTH_SMOKING_YEARS	FALSE	12	2	1	-9999
HEART_RATE	FALSE	44	1	0	-9999

The above table lists variables for which a VALUES entry defines an encoded value (i.e., value=meaning; e.g., 0=male), but that value is not present in the data. While ideally all defined encoded values (i.e., in set V) should be observed in the data (i.e., in set D), it is NOT necessarily an error if one does not.

Interpretation of table column names:
–> `AllVsInD`: Check result communicating if all parsed VALUES entries were detected in the data set (FALSE=no).
–> `NsetD`: Number of values (or levels) detected in the data (e.g., in this example, `LENGTH_SMOKING_YEARS` has 12 unique levels).
–> `NsetV`: Number of encoded value codes detected (e.g., for this example, `LENGTH_SMOKING_YEARS` has two encoded values).
–> `NsetDAndSetV`: Number of occurrences detected in both the data set and the VALUES entries (e.g., for this example, `LENGTH_SMOKING_YEARS` has one of the two encoded values detected in the data).
–> `VsNotInD`: Encoded value not detected in the data (e.g., for this example, -9999 was not detected in either variable).

So this awareness check alerts us to two potential errors. Specifically, -9999 is defined as a missing value code for `LENGTH_SMOKING_YEARS` and `HEART_RATE`, but this code is not detected in the data itself.

```
# Smoking
table(DS.data.B$LENGTH_SMOKING_YEARS)
#>
#> -4444   0.5   1.5     5    10    14    15    25    44    45    50    52
#>    84     1     1     1     1     1     3     2     2     1     2     1
dictionary_search(DD.dict.B,
    search.term=c("LENGTH_SMOKING_YEARS"),
    search.column=c("VARNAME"))
#> # A tibble: 1 x 6
#>   VARNAME              VARDESC                          UNITS TYPE  VALUES ...18
#>   <chr>                <chr>                            <chr> <chr> <chr>  <chr>
#> 1 LENGTH_SMOKING_YEARS How many years has the particip~ years deci~ -9999~ -444~

# Heart rate
table(DS.data.B$HEART_RATE)
#>
#>  38  45  46  47  48  49  50  52  54  55  56  57  58  59  60  64  65  67  68  72
#>   1   5   1   1   2   1   1   2   2   1   3   1   5   1   1   1   8   1   2   2
#>  73  74  75  76  78  79  82  83  85  86  90  91  95  96  98 100 105 107 110 113
#>   1   1   9   3   1   1   1   1   9   2   2   1  13   1   1   1   1   1   3   1
#> 114 115 125 135
#>   1   1   1   1
dictionary_search(DD.dict.B,
    search.term=c("HEART_RATE"),
    search.column=c("VARNAME"))
#> # A tibble: 1 x 6
#>   VARNAME    VARDESC                                    UNITS TYPE  VALUES ...18
```

```
#>   <chr>      <chr>                                      <chr> <chr> <chr>  <chr>
#> 1 HEART_RATE Heart rate measured during blood pressure~ beat~ inte~ -9999~ <NA>
```

Looking at this more closely, we see a missing value code of -4444, not -9999, is being used for `LENGTH_SMOKING_YEARS`, and `HEART_RATE` is a complete variable with no missing data. -9999 could be removed as a `VALUES` entry for those variables and -4444 should added as a `non.NA.missing.value.code` for this function and example data set.

7.1.2.3 Check C: If the user defines a missing value code that is not defined in a VALUES entry (In Set M and Not in Set V).

Table 7: Table Check C: List of variables for which user-defined missing value code(s) are not defined in a VALUES entry.

VARNAME	AllSetMInSetV	NsetV	NsetM	NsetMAndSetV	SetMsNotInSetV
SEX	FALSE	2	1	0	-9999
CUFFSIZE	FALSE	4	1	0	-9999
SOCIAL_SUPPORT	FALSE	5	1	0	-9999
PERCEIVED_CONFLICT	FALSE	2	1	0	-9999
PERCEIVED_HEALTH	FALSE	2	1	0	-9999

Interpretation of table column names:
–> `AllSetMInSetV`: Variable-specific check result communicating if user-defined missing value code(s) are detected as a VALUES entry (FALSE=no).
–> `NsetV`: Number of encoded value codes detected (e.g., in this example, `SEX` has two levels [0=male, 1=female]).
–> `NsetM`: Number of missing value codes defined (e.g., in this example, 1 user-defined missing value code [`-9999`] was defined).
–> `NsetMAndSetD`: Number of occurrences detected in both the user-defined missing value code and data set.
–> `SetMsNotInSetV`: Missing value code defined that was not detected in the VALUES entries (e.g., here -9999).

7.1.2.4 Check D: If a user-defined missing value code is present in the data for a given variable, but that variable does not have a corresponding VALUES entry (M in Set D and Not in Set V).

Table 8: Table Check D: List of variables for which a user-defined missing value code is present in the data for a given variable, but that variable does not have a corresponding VALUES entry.

VARNAME	All_MInSetD_InSetV	setMInDNotInV
CUFFSIZE	FALSE	-9999

Interpretation of table column names:
–> `All_MInSetD_InSetV`: Variable-specific check result communicating if user-defined missing value code(s) are detected in the data for a given variable, but that variable does not have a corresponding VALUES entry (FALSE=no).
–> `setMInDNotInV`: Encoded value codes detected in the data but not in a corresponding VALUES entry.

Note that this check identified a true error! Specifically `CUFFSIZE` has a missing value code in the data, -9999, that has not been defined as an encoded value in the `VALUES` columns. (Funny enough, this was NOT intentional on our part when creating this synthetic data set! Thank you dbGaPCheckup!)

7.1.2.5 Check E: If a VALUES entry is NOT defined as a missing value code AND is NOT identified in the data. ((Set V values that are NOT in Set M) that are NOT in Set D). Passed

In our example here, all VALUES entries that are NOT defined as missing values codes are listed in the data - so our check passes.

However, if there were issues, interpretation of table column names would be as follows:
–> `All_VNotInM_NotInD`: Variable-specific check result communicating if encoded values that are NOT defined as a missing value code are detected in the data (FALSE=no).
–> `setVNotInM_NotInD`: Encoded value codes detected as a VALUES entry but NOT listed as a missing value code and NOT detected in the data.

7.2 Appendix B: Data Report

Next we can run `create_report`, which generates a textual and graphical report of the selected variables in HTML format which will optionally open the report in a web browser. This awareness report is designed to help you catch other potential errors in your data set. Note that the report generated is quite long however, so we recommend that you only submit subsets of variables at a time. In the example below, for speed of rendering, we create the report for variables only in columns 3 through 6. Note that there is an option to plot/report the data split by sex if desired. Specification of missing value codes are also important for effective plotting.

Again, the code below generates a nearly identical output to the `create_report` function, with some additional annotation added here for the purposes of this vignette and ease of interpretation.

```
# Not run as works best when run interactively
create_report(DD.dict, DS.data, sex.split=TRUE, sex.name= "SEX",
   start = 3, end = 7, non.NA.missing.codes=c(-9999,-4444),
   output.path= tempdir(), open.html=TRUE)
```

7.2.1 Summary and plots

```
dat_function_selected(DS.data.B, DD.dict.B,
        sex.split = TRUE, sex.name = "SEX",
        start = 3, end = 6, dataset.na=dataset.na,
        h.level=4)
#>
#> #### AGE - integer
#> Check passed: AGE is integer TYPE and all integers
```

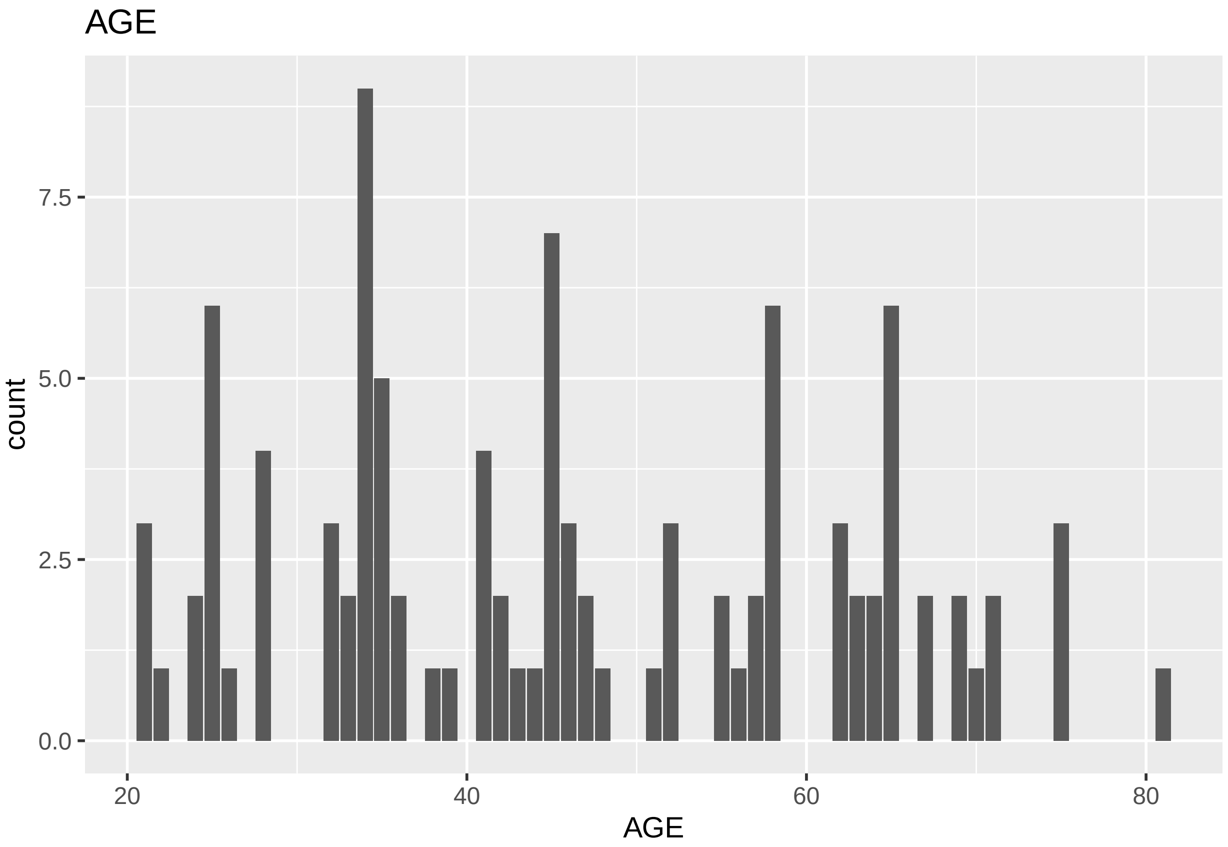
AGE
count
7.5
5.0
2.5
0.0
20
40
60
80
AGE

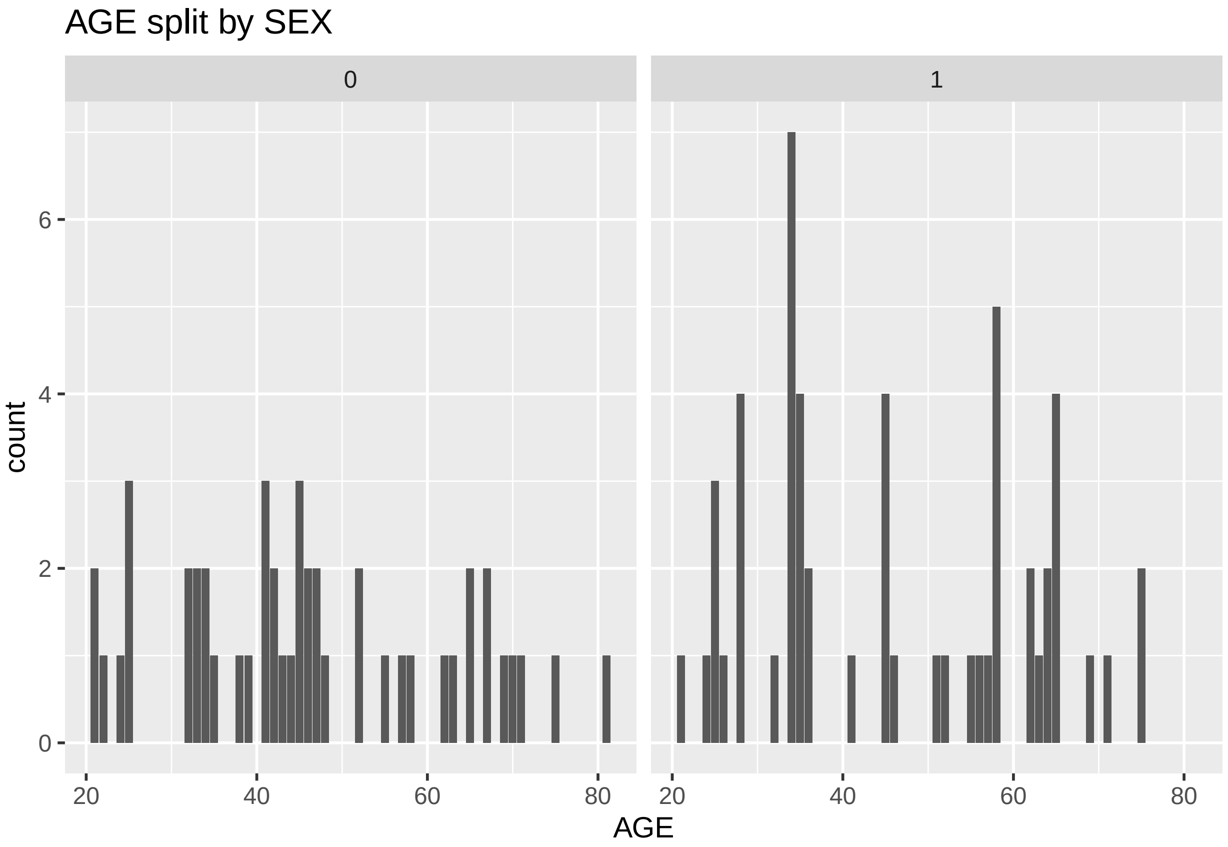
AGE split by SEX
0
1
count
6
4
2
0
20
40
60
80
AGE

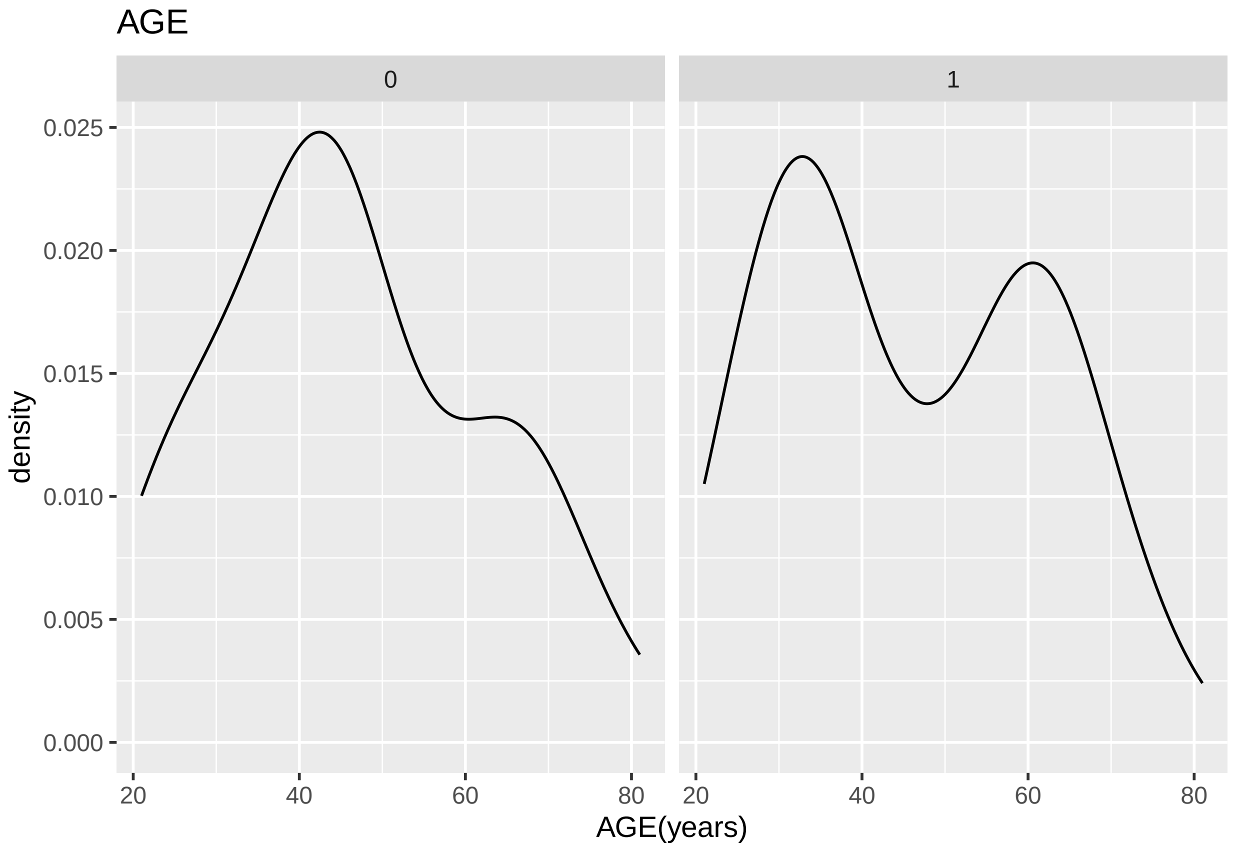
AGE
0
1
0.025
0.020
0.015
0.010
0.005
0.000
density
20
40
60
80
AGE(years)

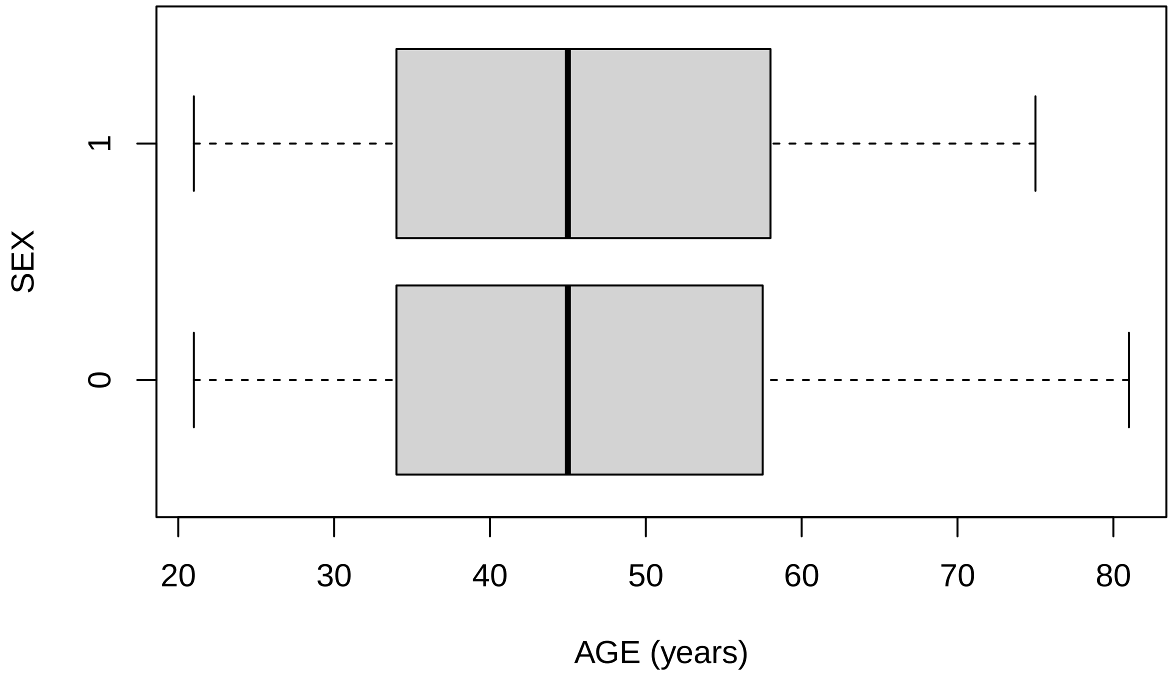


```
#>
#>
#> -
#>  AGE has no missing values.
#>
#>
#> -
#>  AGE has no missing values after mapping missing codes to NA.
#>
#> #### SEX - integer, encoded value
#> Check passed: SEX is integer TYPE and all integers
```

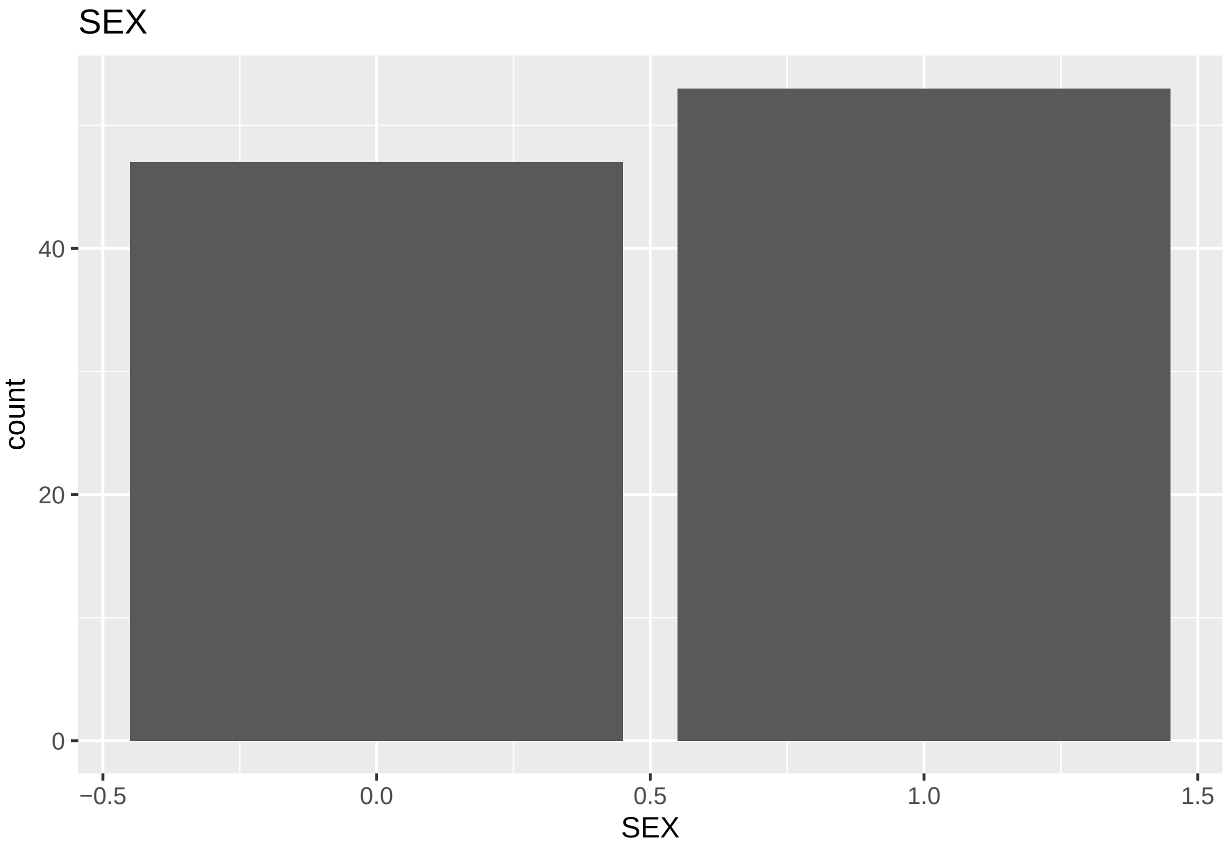


```
#>
#>
#> -
#>  SEX has no missing values.
#>
#>
#> -
#>  SEX has no missing values after mapping missing codes to NA.
#>
#> #### HEIGHT - decimal, encoded value
```

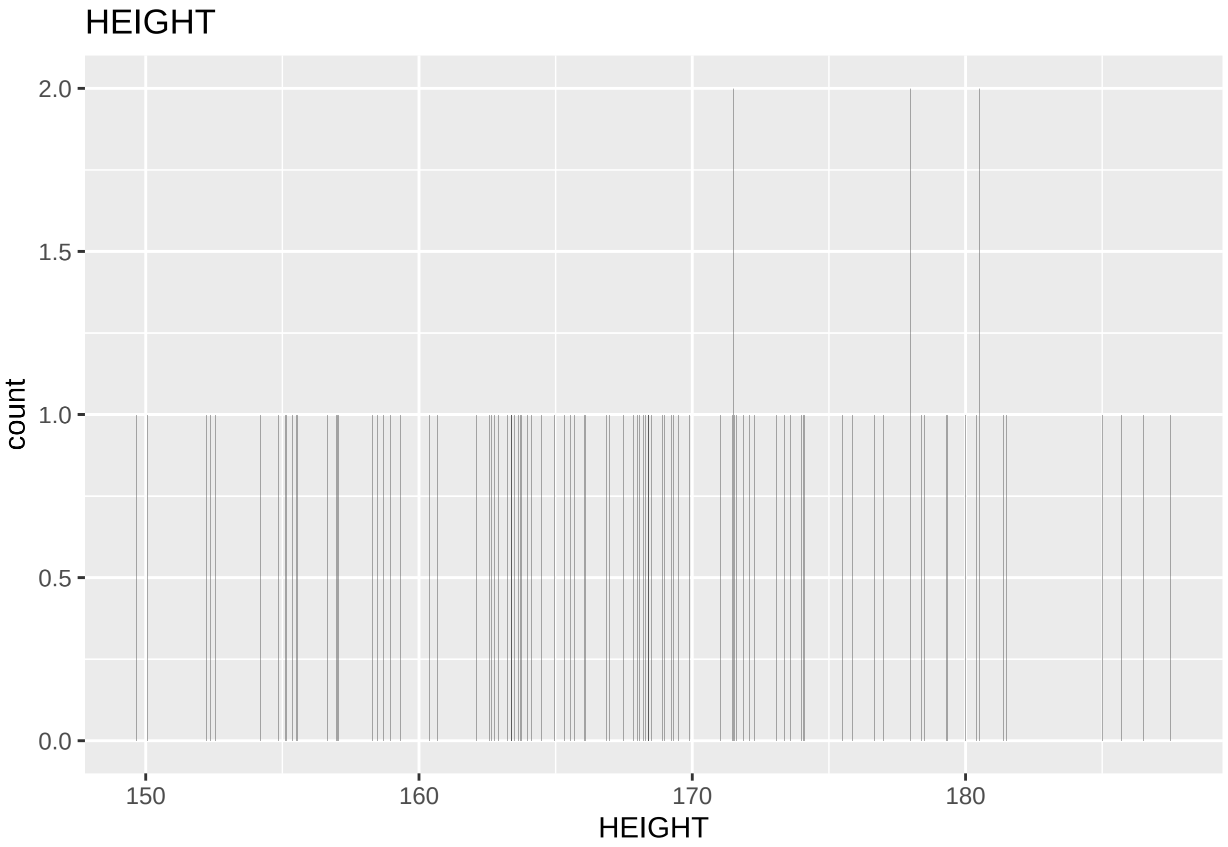
HEIGHT
count
2.0
1.5
1.0
0.5
0.0
150
160
170
180
HEIGHT

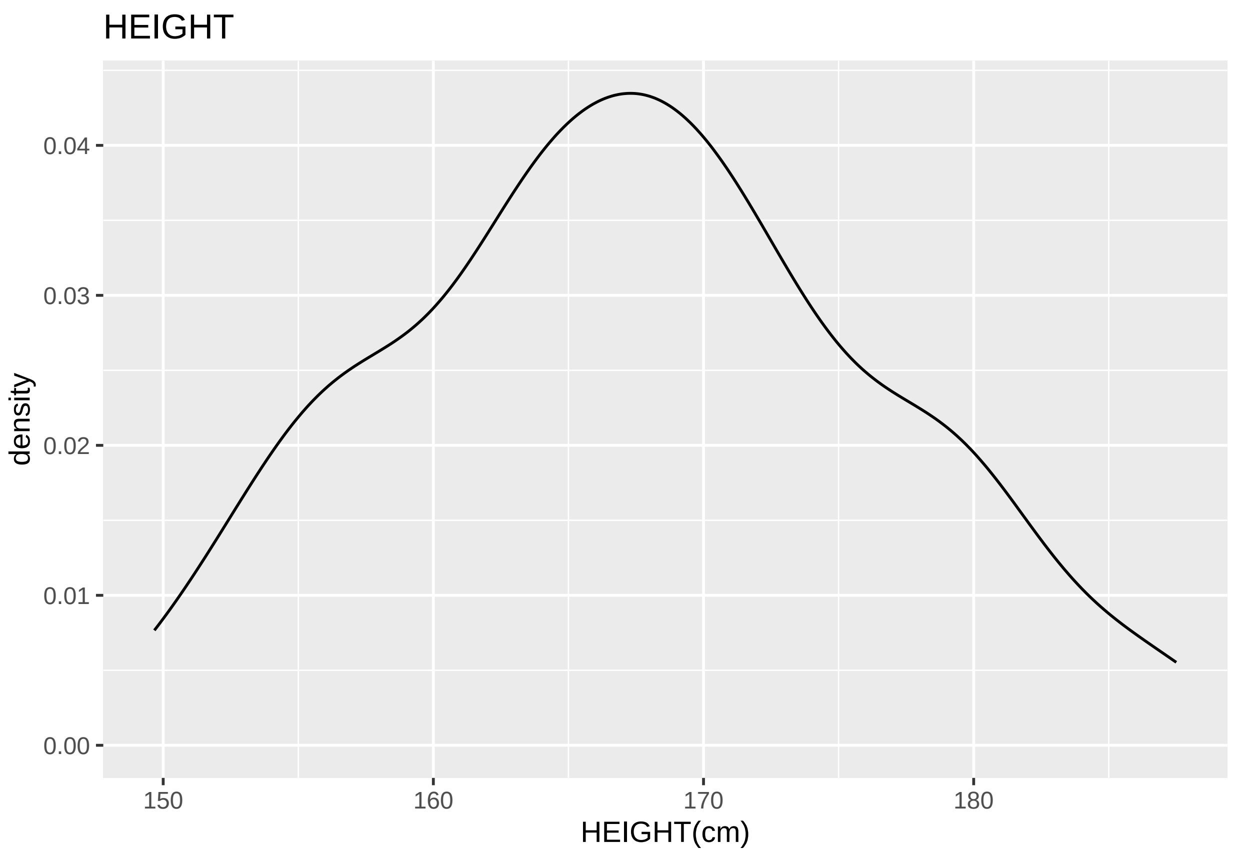
HEIGHT
density
0.04
0.03
0.02
0.01
0.00
150
160
170
180
HEIGHT(cm)

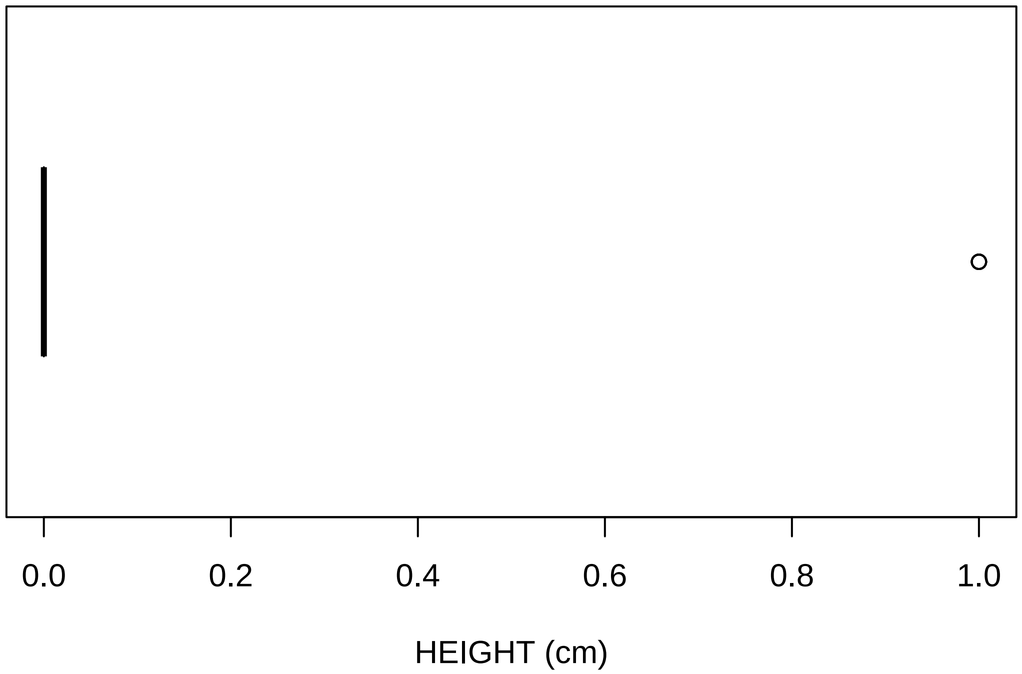
0.0
0.2
0.4
0.6
0.8
1.0
HEIGHT (cm)

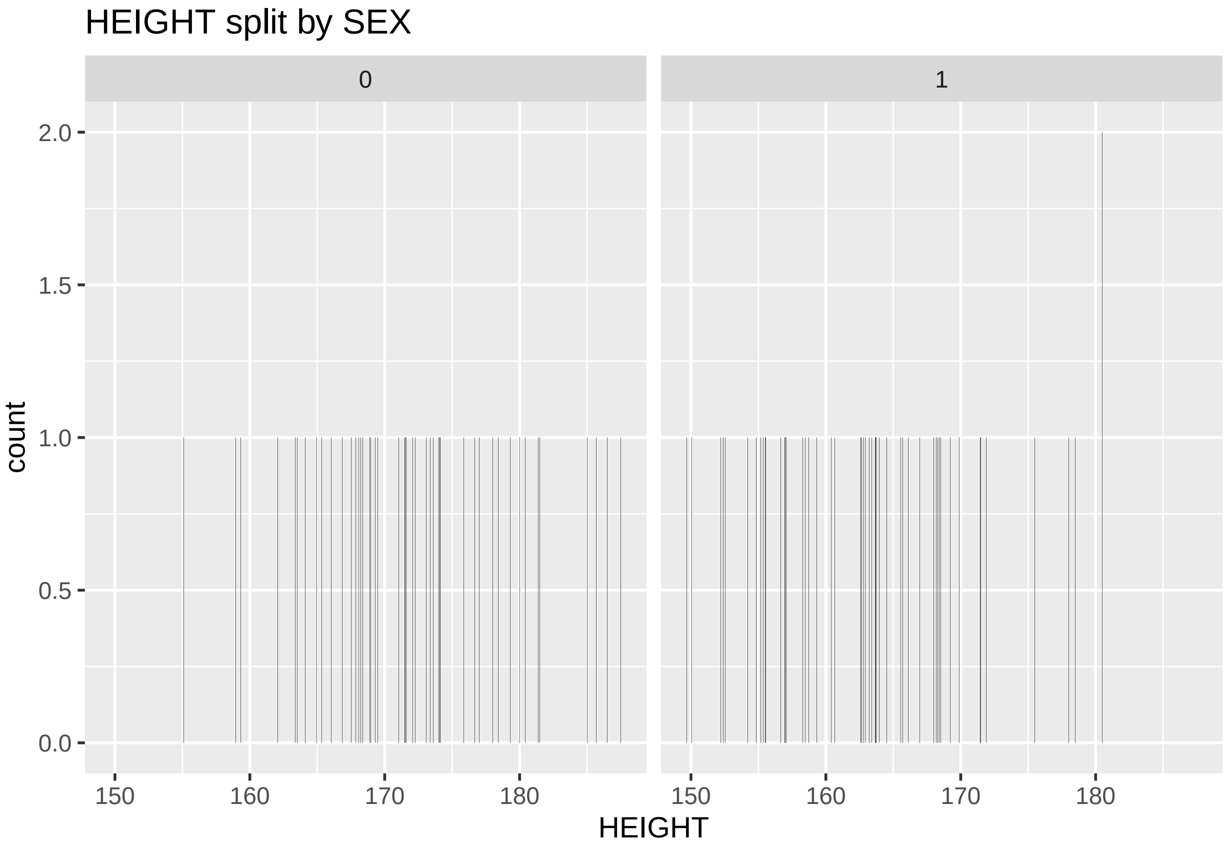
HEIGHT split by SEX
0
1
2.0
1.5
1.0
0.5
0.0
count
150
160
170
180
HEIGHT

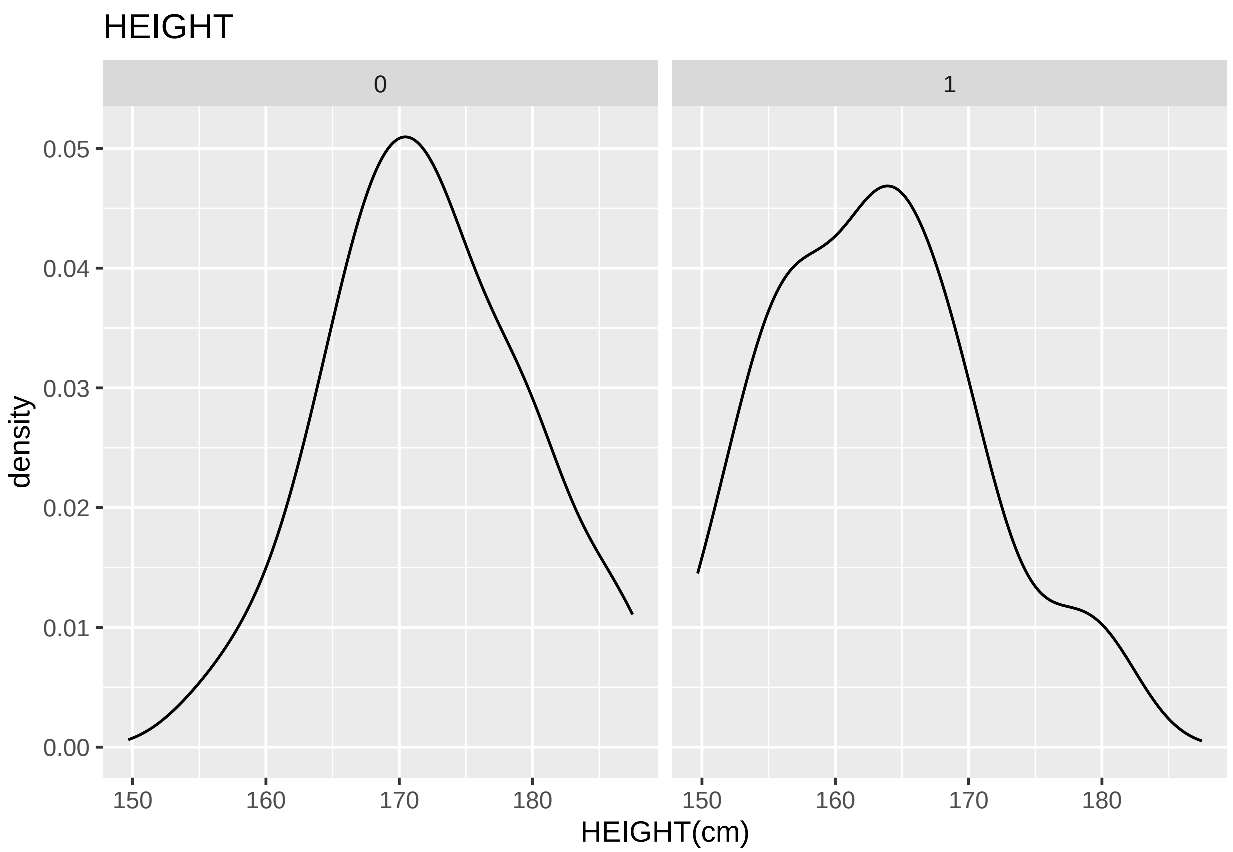
HEIGHT
0
1
density
0.05
0.04
0.03
0.02
0.01
0.00
150
160
170
180
HEIGHT(cm)

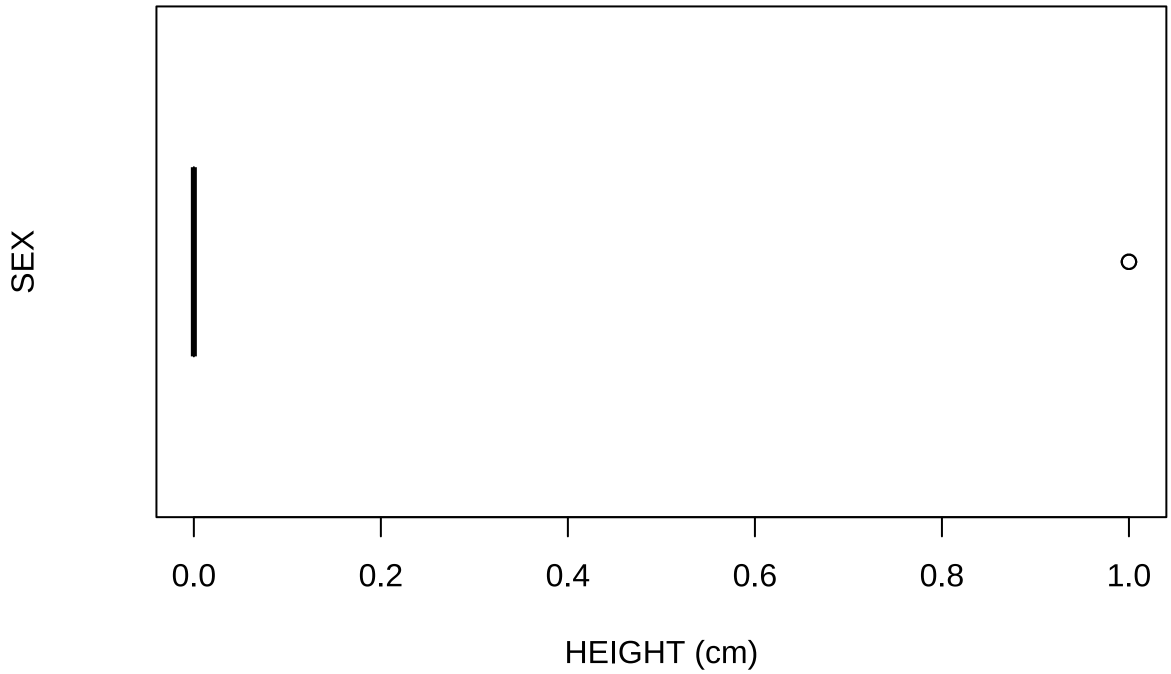


```
#>
#>
#> -
#>  HEIGHT has no missing values.
#>
#>
#> -
#> There are  53  missing values for  HEIGHT  after mapping missing codes to NA.
#>
#> #### WEIGHT - decimal, encoded value
```

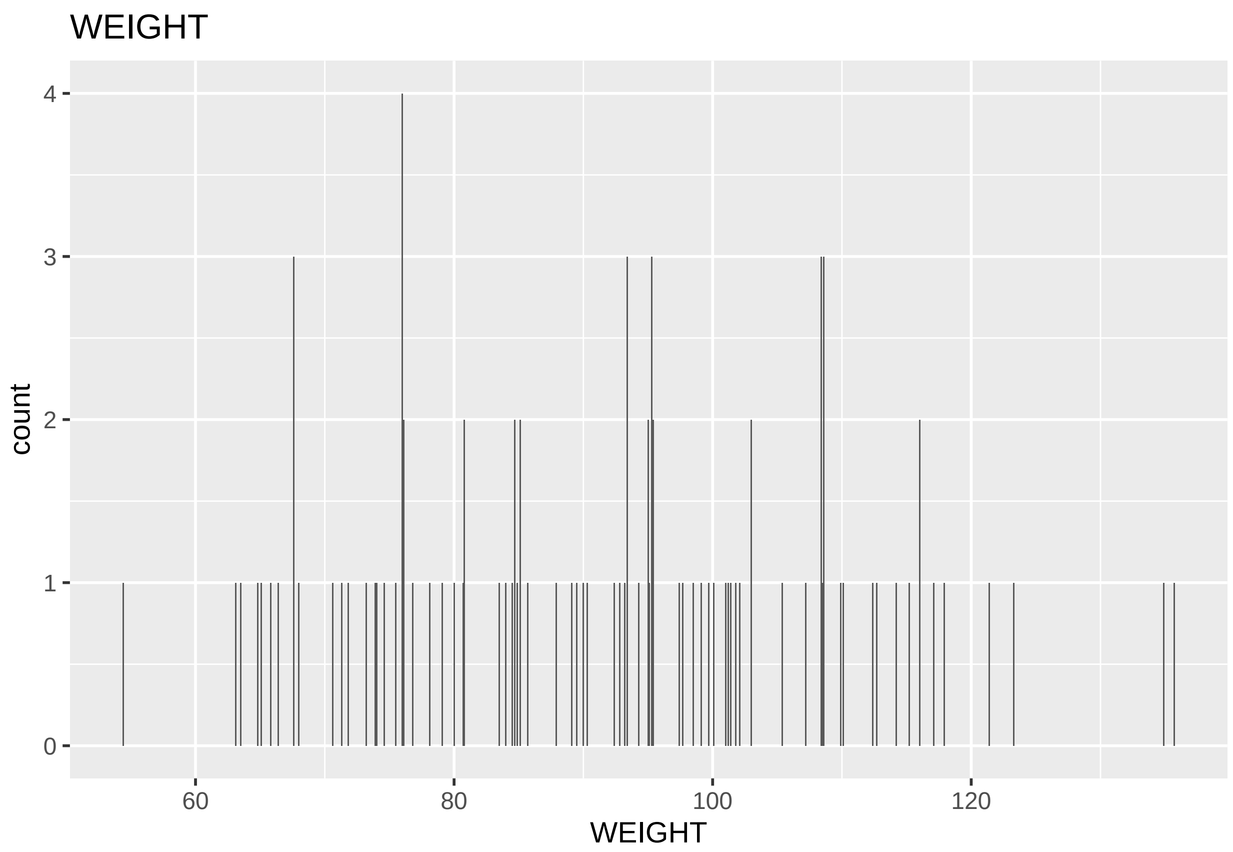
WEIGHT
count
4
3
2
1
0
60
80
100
120
WEIGHT

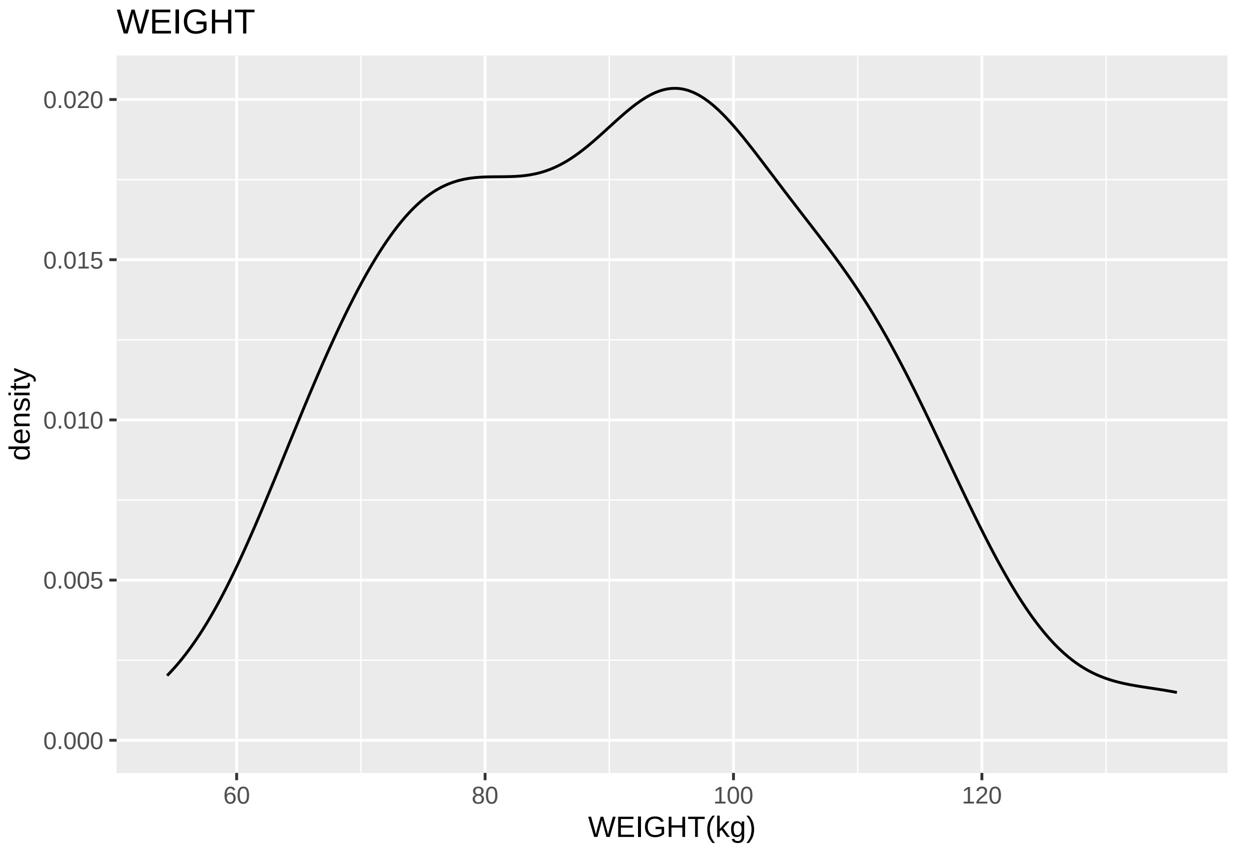
WEIGHT
density
0.020
0.015
0.010
0.005
0.000
60
80
100
120
WEIGHT(kg)

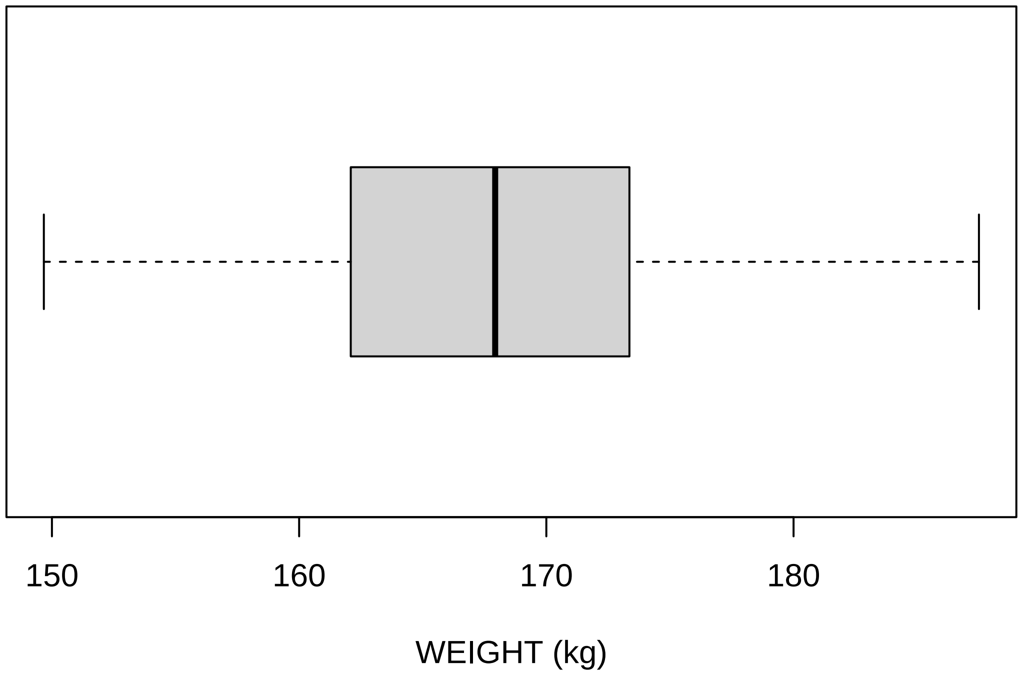
150
160
170
180
WEIGHT (kg)

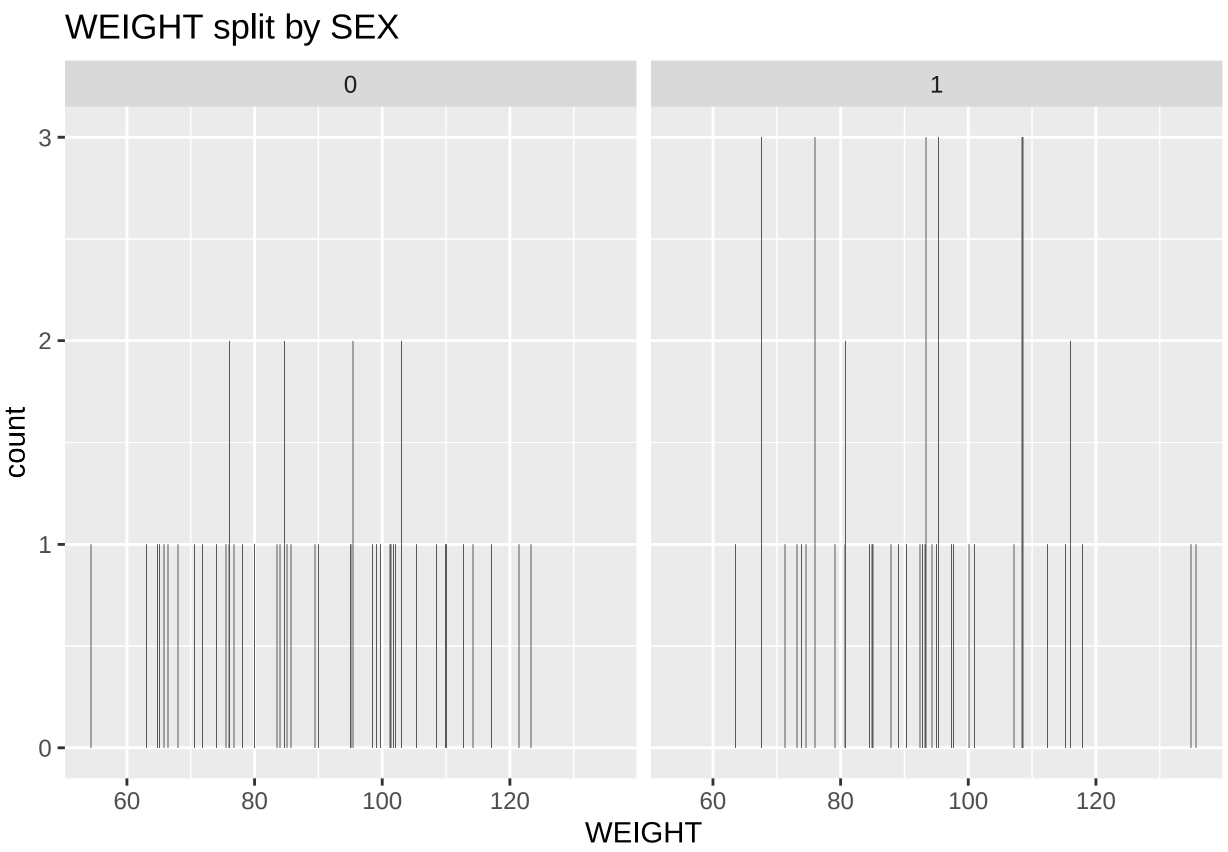
WEIGHT split by SEX
0
1
count
3
2
1
0
60
80
100
120
60
80
100
120
WEIGHT

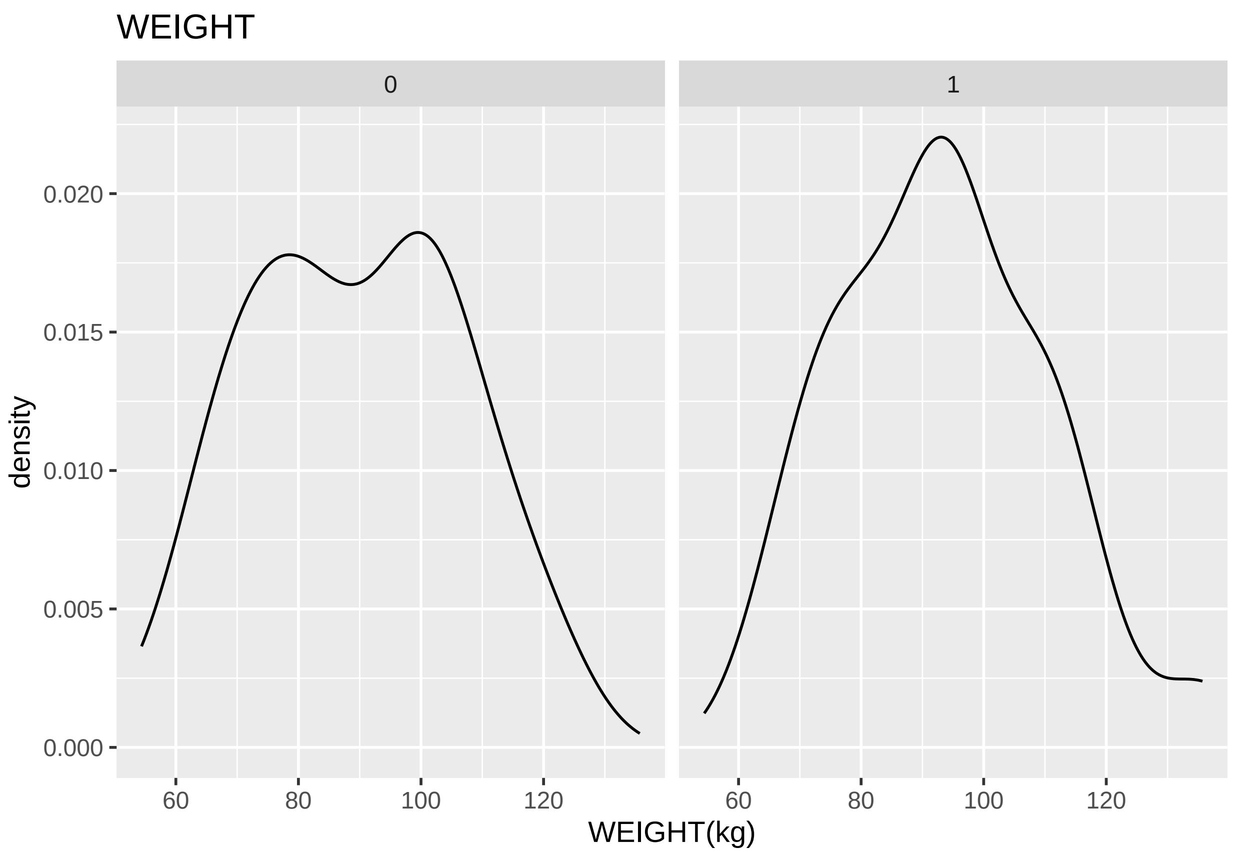

WEIGHT
0
1
density
0.000
0.005
0.010
0.015
0.020
60
80
100
120
WEIGHT(kg)

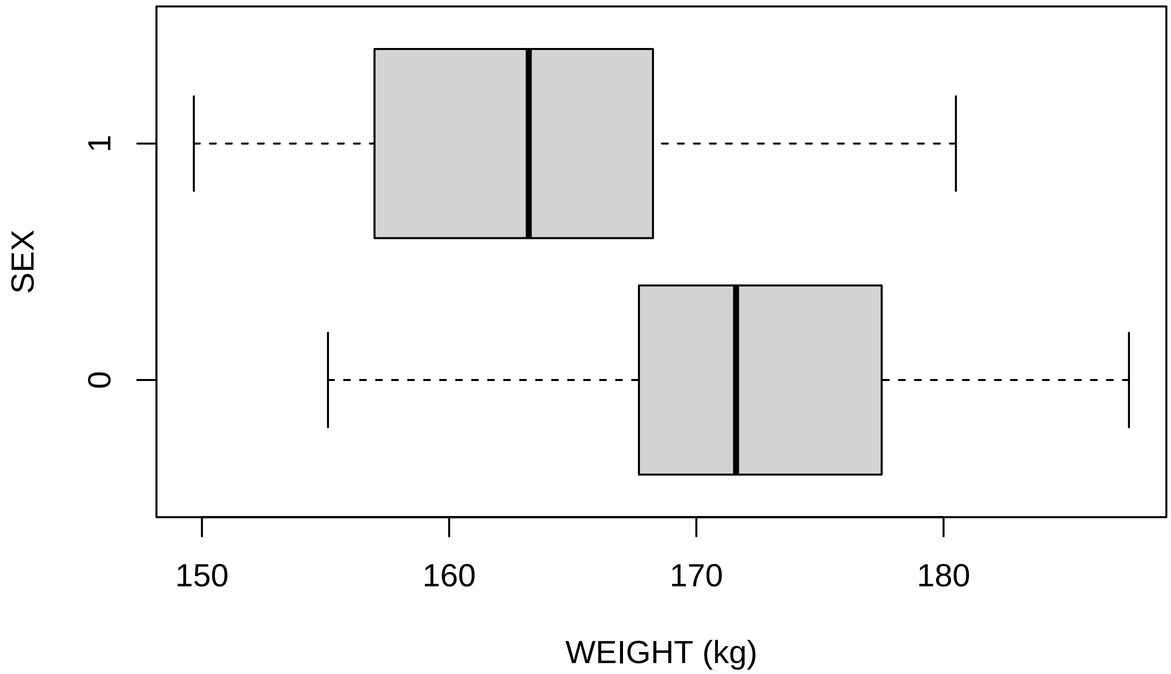


```
#>
#>
#> -
#>  WEIGHT has no missing values.
#>
#>
#> -
#> There are  2  missing values for  WEIGHT  after mapping missing codes to NA.
```

Above we see a full report for variables `AGE`, `SEX`, `HEIGHT`, and `WEIGHT` as well as `AGE`, `HEIGHT`, and `WEIGHT` split by sex. Given the complexity of many data sets, this report was created so that investigators could more easily manually review the data for potential errors (e.g., sex=male appearing in a data of pregnant participants who were all female assigned at birth).

8 Contact information

If you have any questions or comments, please feel free to contact us!

Lacey W. Heinsberg: law145@pitt.edu
Daniel E. Weeks: weeks@pitt.edu

Bug reports: https://github.com/lwheinsberg/dbGaPCheckup/issues

9 Acknowledgments

This package was developed with partial support from the National Institutes of Health under award numbers R01HL093093, R01HL133040, and K99HD107030. The `eval_function` and `dat_function` functions that form the backbone of the awareness reports were inspired by an elegant 2016 homework answer submitted by Tanbin Rahman in our HUGEN 2070 course 'Bioinformatics for Human Genetics'. We would also like to thank Nick Moshgat for testing and providing feedback on our package during development.
